# Understanding UK policymakers’ evidence needs through policy questions

**DOI:** 10.1038/s41598-025-05911-3

**Published:** 2025-07-02

**Authors:** Magda Osman, Nick Cosstick

**Affiliations:** 1 Judge Business School, University of Cambridge, Trumpington Street, Cambridge, CB2 1AG UK; 2 Leeds Business School, University of Leeds, Maurice Keyworth Building, Woodhouse, LS2 9JT Leeds, UK

**Keywords:** Evidence-based policymaking, Public policymaking, Policy inquiries, Science-policy exchanges, Psychology, Human behaviour

## Abstract

The present mixed methods study used UK policymakers to answer the following: (1) are there common topics for which evidence is requested over time (2019 to 2023) that cut across government departments or agencies, and (2) is there a preferred style in the way evidence is requested? Three separate datasets of policy questions (*n* = 3260) posed by UK policy makers to academics were coded by a combination of humans and an algorithm and then analysed. First, of the 7 recurring topics identified (Climate and Environment, Defence and Security, Economy, Health, Information Technology, Social Welfare, Technology), Economy (27%) was the most featured across all policy makers across all 5 years. Climate and Environment showed the sharpest rise over time (16–38%). Second, of 7 styles of questions, procedural (33%) was the most common, which means addressing “how to” (e.g. measure, intervene, prevent) type questions. In the qualitative interviews policymakers reported gaining the most from an exploratory rather than a goal-specific approach during one-to-one interactions with academics. Also when having their assumptions challenged this helped to expand the way they thought of policy issues that they were currently addressing. This UK test case shows the value of focused iterative policy-academic exchanges and could be a way to enhance evidence-based policymaking initiatives.

## Introduction

In the present study, we use the UK as a test case to examine approaches to evidence-based policymaking (hereafter EBPM) by focusing on what evidence requests policymakers present to academics. As will be discussed later in more detail, in the UK civil service, requests for evidence can take the form of a public record (e.g. Areas of Research Interest) which allow for a detailed analysis of the kind conducted presently. Moreover, because evidence requests come from several different government departments and agencies, we can use public data sets to examine the following: (1) are there common topics for which evidence is needed that cuts across departments and agencies, (2) is there a common style in the way the evidence is requested? Answering these questions inform the research community where there are continuing evidence gaps that academics could help plug, and where to avoid replication of effort because the same evidence gaps repeat in several policy domains and so can be targeted in the same way. From a policymaker’s perspective, an analysis of the recurring topics and styles of evidence requests can help identify where there are opportunities for more cross-governmental work, to more efficiently utilise the evidence provided, and to identify what might be the source of the problem if the exact same evidence requests are made repeatedly year on year. Regarding cross-governmental work, many complex policy problems (e.g., regulation of AI) do not easily sit within a single department or agency, and so, just as the demands for multidisciplinary research increase, so too is the case for cross-governmental work. The present study can also help to expose this because of the bird’s eye view it takes through the analysis of questions based on the recurring topics of questions that span across departments and agencies. In addition, the present study explores in depth in a series of structured interviews the views of policymakers based on their experiences of exchanges with academics as route to EBPM, and exposes some of the contextual factors that inform the way in which they design policy questions they pose to academics.

The demand for evidence in public and social policymaking may be as high as it has ever been since EBPM gained popularity following its inception in the 1980s^1^. EBPM is widely debated in terms of its definition^2^, with a dedicated field examining the processes involved in academic engagement through which evidence is translated in ways to inform policymaking^3^. Evidence means information (e.g. qualitative interviews, case studies, surveys, experimental data, reviews, meta-analyses) that is used to support a claim, argument, or hypothesis^4^. It is worth noting that what constitutes evidence in the world of policymaking can also vary hugely from what is typically construed as evidence in scientific/academic circles. For instance, in policymaking, expert opinion is also deemed evidence, which is not the case in academic circles. In broad terms, EBPM can be construed as the provision of evidence for the purposes of informing decisions about agenda setting, policies, programs, and projects carried out by public policy organizations^5–7^. The armory of evidence that policy professionals have at their disposal is available to be utilized for whichever needs exist—while taking into account constraints regarding the application of that evidence and the timescale in which it is needed.

When it comes to accessing evidence, academia is a key stakeholder in EBPM. It is often one of the first ports of call that policymakers consider—accessing research in the form of rapid reviews, meta-analyses, modelling, and empirical findings. One way of ensuring that academia can make valuable contributions to EBPM is to understand common themes in the topics and style of evidence being requested, which can often take the form of policy questions^8–10^. The reasoning behind this is that tracking the trends in the topics for which requests are made, and having a good idea of the common style of evidence requests, makes two things possible. When new research and technological discoveries are made, if they have policy implications, then researchers can anticipate questions that will come from policymakers. If applied researchers—such as those examining interventions that improve outcomes in social policy domains—know how to tailor the presentation of evidence to align with the style of requests that policymakers make, this would facilitate a better uptake of their evidence^8,9,11^.

Policymakers and policy institutions are the key stakeholders of EBPM. There are many illustrations of the efforts taken to embed EBPM as standard practice in public policymaking organizations. For instance, the Foundations for Evidence-Based Policymaking Act of 2018 was enacted by the US government in 2019. What Works Networks—which were established in the UK Government in 2013 and are still running—operate with the principle aim of making available the best evidence to inform and support decision-making in government and other public sector organizations. In the EU, the Joint Research Centre is specifically set up to provide “independent, evidence-based knowledge and science, supporting EU policies to positively impact society” (Joint Research Centre, 2024). To put things in context, EBPM is a key process for informing the way in which public policy agendas are set, how they are addressed—how interventions are trialed, and how the policies are monitored and evaluated^12–19^. In essence, the objective of EBPM is for policy professionals to make decisions that best reflect the scientific evidence showing what, how, and why a policy instrument works.

Questions are standard tools by which evidence requirements are signaled^8–15^, this is because they are diagnostic of where^14–23^ evidence is needed, and the kind of evidence needed to address those gaps^8–10,12^. For instance, the UK government has developed an EBPM initiative referred to as Areas of Research Interest (ARIs) where government departments, government agencies, and arm’s length bodies regularly publish topics and specific questions on the Government Office for Science website. ARIs acts as an invitation to researchers to contribute their work in helping to answer the questions, or even helping to inform new ones. Several studies have investigated the relationship between the topics that frequently come up in ARIs and the funding initiatives that support applied research, how ARIs can facilitate cross-governmental sharing of findings, and the reasons for reservations that researchers have in supporting this kind of initiative^16,17,22,23^. While the enthusiasm to serve the public good by generating evidence that would be of value to policy is what drives many academics to consider EBPM schemes, they struggle to understand how their research could contribute to informing policy. In addition, researchers often lack experience in interacting with policymakers or engaging with policy related source materials, which contributes to skepticism as to how their work could meaningfully contribute to^22^, or could be used to inform policy^23–25^. To date, the work reporting on potential barriers in academic engagement with EBPM converges on several recurring factors. The barriers typically come from either unfamiliarity with policymakers and policymaking, limited incentives, and perceived misalignment between academia and policy on the concept of evidence^11,23,25–30^.

To answer the novel questions posed in this study, a recently developed classification system for identifying patterns in policy-related inquiries was used: the ‘Taxonomy of Policy Questions^8,10^ (hereafter TPQs). TPQs categorizes questions by type (“style”) using the concept of the structure of the information sought based on the anticipated answers that the questioner has in mind^12,19–21^ (see Table 1). TPQs includes 7 styles of questions that invite particular types of answers (Verification, Comparison, Forecasting, Procedural, Example/Explanation, Asserting Value Judgments, Causal Analytic). TPQs organizes the questions into a super-ordinate category of unbounded and bounded questions, where the difference between the two is based on signaled constraints on the answers invited^18–21^. Unbounded questions are essentially open-ended because they don’t have a predetermined range of options that an answer should address (e.g. How can AI be used in healthcare? ). Bounded questions signal specific restrictions around the answer that is invited (e.g. What are the costs and benefits of using AI in healthcare? )^8–10^.

**Table 1:** Outline of question types: style, outcome orientation, and use orientation.

Question type	Category		Abstract specification/concept explanation	Example
Style	Super-ordinate	Sub-ordinate		
	Bounded answers	Verification	Is it the case that X is here? Did X event occur? Are Xs more inclined towards y? Is X a viable version of Y?	Do groups generally make better decisions than individuals? What are the justifications for using groups to make decisions?
		Comparison	What are the strengths and weaknesses of X? What are the costs and benefits of implementing X?	What are the costs and benefits of decision-making through groups over individuals?
		Forecasting	Which areas would you foresee improving in the next 10 years? How likely is it that X will be popular in the future?	How will group decision-making shape action in the next 10 years?
	Unbounded answers	Example/ Explanation	Which X is more like Y? What would be a case where Y is like X? How does X work?	Can you illustrate situations where groups make better decisions? How does group decision-making work?
		Causal analysis	What are the barriers that will prevent X from occurring? What are the effects of X if it is implemented now?	What happens if groups end up making the wrong decisions? What brings about the need to use groups in situations of adversity?
		Procedural	How can we use X to make Y better? What would need to be incorporated to ensure that X is produced? In what way can we measure X so that it can later be used to support y?	What strategies can organization X implement for group decision-making to occur? What are the methods and strategies by which individuals can be encouraged to work in groups?
		Asserting value judgments	How should the infrastructure available be used to produce x? How should X respond to y?	Why do you think groups are the best way to make decisions?
Outcome orientation	Preventative perspective		Seeks answers regarding how to prevent negatively valanced phenomena.	What actions should the UK take to mitigate climate disasters in the global south?
	Generative perspective		Seeks answers regarding how to bring about positively valanced phenomena.	How can we strengthen local implementation of sustainability goals and empower local communities to make more sustainable choices?​
Use orientation	Informing-policy perspective		Concerns the search for evidence to inform a specific policy topic.	What will the policy impacts of an increasingly accessible Arctic be for the UK’s Climate and Energy Policy?
	Evidence-search perspective		Concerns the search for evidence to inform general purpose evidence search.	How can we make more effective use both of existing and new data and analytic methodologies to generate the intelligence to support our decision making to ensure that hazard identification and consequence models are validated for new technologies and new ways of working?

Osman and Cosstick^8^ utilized a dataset of 2927 questions posed by over 400 predominantly UK policymakers to researchers over a 10-year period to generate TPQs—a refined version of Graesser et al.’s taxonomy^18,19^. The findings, based on the application of TPQs, reveal that the most frequent question style deployed by policymakers was the Procedural style^8–10^: inviting answers informing how to achieve a certain goal in an instrumental way. However, when researchers address policy-related issues in an impactful way, recent work reveals they typically answer questions designed to verify whether or not something is the case (Verification), causally analyze a phenomenon (Causal Analysis), or explain a phenomenon often through example (Example/Explanation)^9^. This suggests a mismatch between the common style of question researchers address, and the ones that policymakers want addressing. Do policymakers and researchers agree on what style of policy question is of good quality? Even if they aren’t aligned in what preferred style of question they generate to request evidence or to provide evidence, they may agree on what a good policy question looks like. In answer to this, an empirical study using different audiences (academics, policy makers, public) and asked them to rate policy questions on the topic of anthropogenic climate change, the questions were exemplars of the 7 styles identified in TPQ^10^ which were short [10-15 words] or long [30-38 words]. All three types of audience showed a high level of agreement that procedural style questions were judged the best according to quality of communication, neutrality, and overall goodness (incl. persuasiveness, worth asking, stylistically good), with a strong preference for them to take a long form. But that for short form question, verification style questions were judged as the best on dimensions of quality. These findings suggest that while researchers design questions that inform policy differently to the requests for evidence from policymakers^8^, they do align with policymakers as to which style of question is considered best on various dimensions of quality.

Where previous work^8–10^ focused on a narrow data set of policy questions from UK policy makers and empirically examined ratings of quality of policy questions, the topic of the question has yet to be examined in detail. As yet, we do not know whether there is an overlap in the topics for which evidence is requested across different government departments and agencies, and if this happens consistently over time. A question’s style is distinct from its topic^18–21^, and the latter is of critical focus for present study, along with other factors not previously explored, such as trends in topics over time across a range of different data sets of policy questions. For example, consider the questions ‘how does inflation work?’ and ‘how can inflation be decreased?’ both have the same topic: inflation. Yet, they ask for differently structured evidence regarding inflation: an explanation of inflation versus ways to lower it^8^. Therefore, the present study also investigates whether there are patterns in the requests for evidence by topic and by style of question.

As discussed at the start of this section, looking for patterns in the evidence needs of policymakers in the pursuit of EBPM has practical implications for those providing the evidence (i.e. most often the scientific academic community) and those utilizing the evidence (i.e. the policymakers themselves). If one of the goals of EBPM is to make effective use of scientific evidence to optimize the way in which a policy issue can be solved, then how the evidence is requested matters. The requests for evidence become a diagnostic tool for exposing misalignment between the way a problem is construed by the policy makers, and how the same problem is decomposed and examined scientifically^25^. To put this starkly, there is a risk that policymakers frame a question in mind of anticipating what the solution should be to it, this in turn means the problem can be mischaracterized, and the solution is likely to be inappropriate. A reason for this is because the evidence needs of policymakers are typically styled in practical ways that involve asking how to implement a measure, interventions, communications strategy. By contrast, for instance, the scientific community is oriented towards understanding the underlying mechanisms that might bring about the policy problem itself. As mentioned, these different ways of asking questions leads to misalignments in how a policy problem decomposed is supposed by recent evidence from Osman and Cosstick^8–10^.

To achieve the various aims outlined in this section, the present study conducted a detailed analysis of policy questions gathered from three sources, where it was possible to compare the questions over the same period (2019 to 2023). Two of these sources were questions generated by those working in public policy organizations who have taken part in one of two UK-based fellowship schemes at (1) the University of Cambridge’s Centre for Science and Policy^8^ (CSaP) or (2) Capabilities in Academic Policy Engagement (a partnership between five universities [Universities of Cambridge, Manchester, Northumbria, and Nottingham] based at University College London; https://www.cape.ac.uk). Both CSaP and CAPE are knowledge brokerages: they act as intermediaries between policy and academia, so that policy is optimized based on access to the best quality evidence available. As a two-way function, the translation also works in the direction that serves the scientific community. For researchers who are motivated to generate findings that have societal impact the brokerage can increase the opportunities researchers have to do this through engagement with policymakers^31^.

The third source are ARIs: questions generated by UK-government departments, agencies, and public bodies (https://www.gov.uk/government/collections/areas-of-research-interest). The former (i.e CSaP, CAPE) questions are often generated by individual policy fellows themselves rather than collectively in teams. Unfortunately however demographic details of individual fellows are not available to be analyzed in the present study because, in most cases, they wished to remain anonymous. In contrast, the latter kind of questions from ARIs are generated by teams working in public policy organizations, which also means that demographic details of those generating the questions could not be provided for analysis. Nonetheless,  when combined, these sources of data at least give an indication of the kinds of questions generated for two different projects: narrow (targeted) and broad (signaling) evidence search.

Given the limitations in developing a clear profile of the individuals involved in generating policy questions, and as a way to better understand the thinking which informs the formulation of the questions, a qualitative study was conducted. Policymakers’ views regarding what can be learned from science-policy exchanges were gathered through structured interviews with some of the CSaP and CAPE policy fellows.

## Results

Prior to conducting the analysis of topic and style of questions, we examined whether the three different data sets could be collapsed into a single data set for analysis. We found that there were no substantive differences between the data sets (see Methods section), and therefore collapsed across the three. Not that the supplementary materials provide a detailed breakdown of which government departments and agencies the questions came from, and this detail included in the raw data files (https://osf.io/h7duc ). This section is organized by first focussing on the quantitative data by examining the questions by common topics and style to explore trends. We then present the findings from the qualitative study.

### Topic analysis overall

Based on a detailed analysis of the questions using six independent coders, the entire dataset (*n* = 3260) collapsed across three different sources (CSaP, CAPE, ARIs) was used to identify the most commonly occurring topics. A total of 7 were identified (Information Technology, Health, Economy, Climate and Environment, Social Welfare, Technology, Defence and Security) (see Table 2). Each individual question was coded as associated with a specific topic based on the frequency of keywords associated with the topic; this avoided any possibility of double counting and meant that the percentages are accurately reported. Overall, the most common policy topic inquired about was the Economy, and the least frequently occurring topic overall was Social Welfare. Regardless of the frequency of topics appearing in the questions, the length of the question for each topic was approximately the same (*M* = 20.32, *SD* = 10.55; range 3 words to 128 words).

**Table 2:** Basic descriptives of questions by theme and type overall across the 5 years.

Theme	% of all Qs (*n* = 3260)	Mean (SD) word length	Least common style of Question	Most common style of Question	Generative (G) vs. Preventative (Pr)	Informing Policy (IP) vs. Evidence Search (ES)
Information Technology	17.33 (*n* = 453)	*M* = 23.11 *SD* = 12.60	Comparison 2.65%	Procedural 33.98%	G = 38.41% Pr = 13.81%	IP = 24.78% ES = 8.50%
Social Welfare	11.60 (*n* = 378)	*M* = 23.15 *SD* = 12.50	Forecasting 1.32%	Procedural 33.33%	G = 44.44% Pr = 21.69%	IP = 22.49% ES = 8.73%
Climate and Environment	20.83 (*n* = 679)	*M* = 22.96 *SD* = 11.78	Comparison 2.65%	Procedural 36.43%	G = 41.68% Pr = 18.71%	IP = 18.85% ES = 25.18%
Economy	27.30 (*n* = 889)	*M* = 21.59 *SD* = 11.32	Comparison 3.49%	Procedural 28.94%	G = 42.63% Pr = 15.41%	IP = 21.37% ES = 7.42%
Health	16.20 (*n* = 528)	*M* = 22.50 *SD* = 12.19	Comparison 2.08%	Procedural 31.44%	G = 46.97% Pr = 21.21%	IP = 14.78% ES = 9.85%
Technology	20.00 (*n* = 650)	*M* = 22.49 *SD* = 12.14	Comparison 4.47%	Procedural 33.90%	G = 43.08% Pr = 15.23%	IP = 25.54% ES = 23.85%
Defence and Security	13.92 (*n* = 454)	*M* = 20.87 *SD* = 9.92	Comparison 2.86%	Procedural 34.36%	G = 53.96% Pr = 19.16%	IP = 29.30% ES = 13.00%

**Fig. 1:**
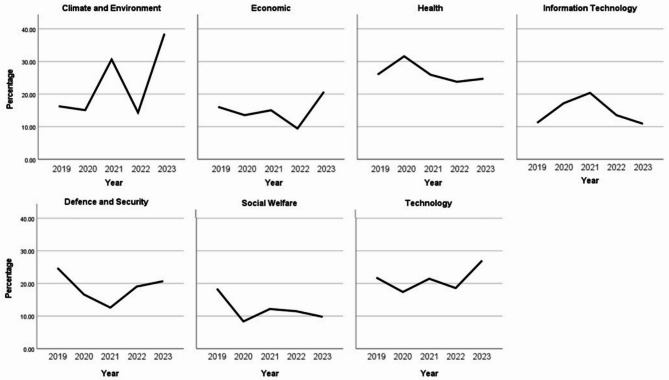
Proportion of questions by theme and by time (5 years 2019 to 2023).

**Fig. 2:**
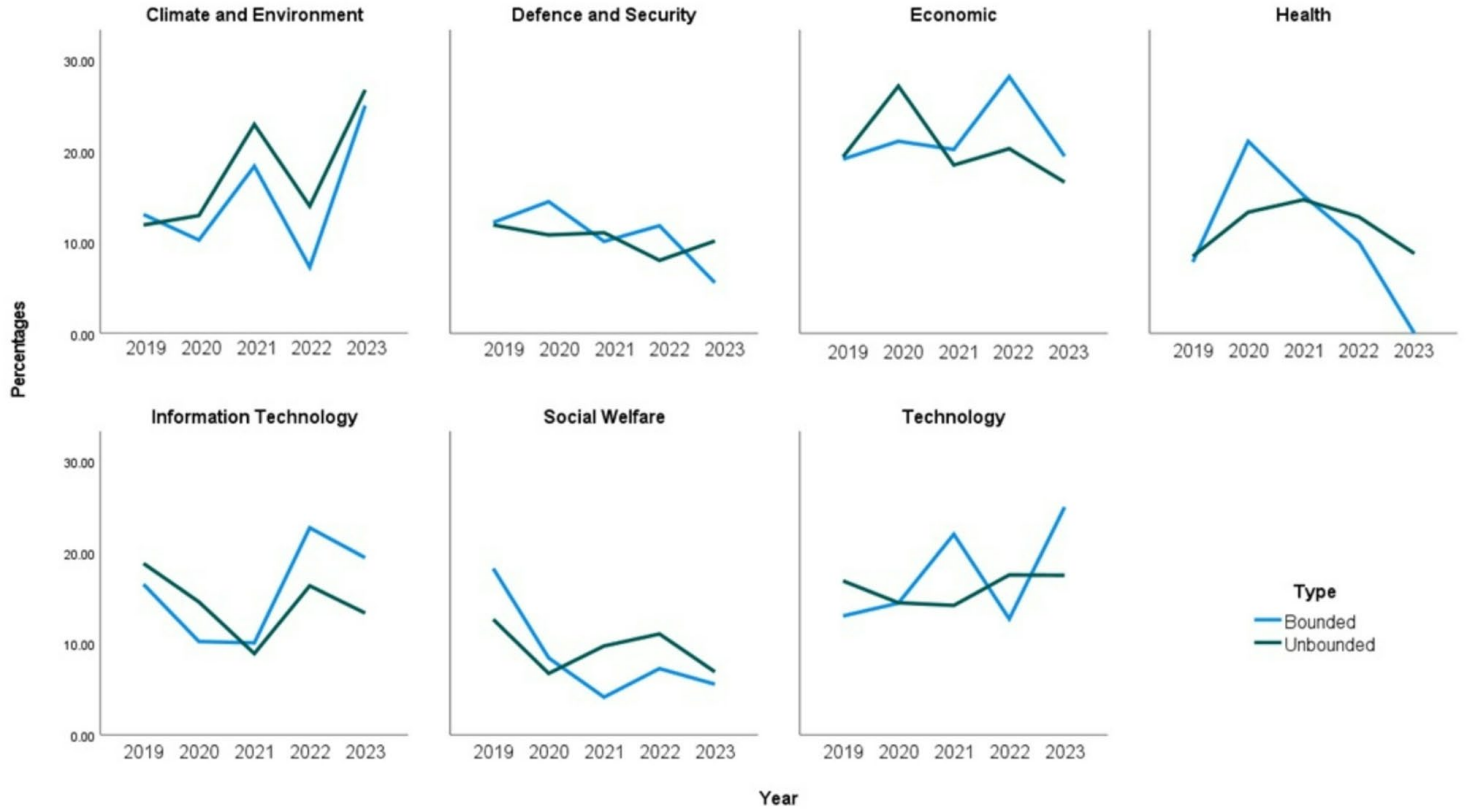
Pattern of distributions of theme by super-ordinate categories from 2019 to 2023.

Two figures are presented concerning the trends in topic over time. Figure 1 shows the percentage of questions by theme over time. Figure 2 shows the percentage of questions by theme and super-ordinate category Bounded vs. Unbounded) over time. The figures indicate that the Climate and Environment policy topic appears to have the sharpest rise across the five years, with moderate increases for Information Technology, Technology, and Defence and Security. We also see moderate decreases, particularly for the past three years, for policy topics concerning Social Welfare and Health.

### Relationship between topic and style of questions

Consistent with previous findings^8–10^, overall, across topic and year, Unbounded questions were more common than Bounded questions—with a ratio of 6:1. In each topic, approximately one third of the questions were Procedural (Unbounded) (range 28.94–41.11% across topics), again, consistent with previous findings^8–10^. All but one of the 7 topics had the Verification style as the most typical of the Bounded category—though this was approximately one twelfth of all questions per topic (range 5.87–9.79% across topics). The only topic which differed was Climate and Environment, where the most frequently generated Bounded question was Forecasting (5.31%).

To examine general trends by topic and style of question over time, the descriptives presented in Fig. 2 highlight three main patterns. First, Climate and Environment topic rose more sharply over time for Bounded compared to Unbounded questions. Second, while Unbounded questions fluctuated over time across the topics, these changes were less pronounced overall compared to the sharper rises and falls observed for Bounded questions. Third, Bounded questions occurred less frequently overall. It is the general open-ended nature of Unbounded questions—they do not constrain the style of answers invited in the same way that Bounded questions do—which leads to their popularity^8^. Moreover, Unbounded questions are judged as suitable, compared to Bounded questions, for domains in which the problems are complex because they invite more details in a response as was demonstrated in previous empirical work examining ratings of quality of policy questions^10^.

### Further analysis

During the coding of the questions, further insights were gained. The six coders drew attention to other possible ways of classifying the questions beyond style and topic. It was clear that the same topic and question style varied in its framing. That is, a style of question might still cover the same topic, but there were multiple ways in which it could be approached. One such distinction in framing concerns the outcome one is aiming at (‘outcome orientation’; see Table 1). On the ‘preventative perspective’, the answer’s perspective should concern the prevention of a possible outcome. Thus, preventative questions seek answers regarding how to prevent negatively valanced phenomena (e.g. the extreme effects of climate change). On the ‘generative perspective’, the answer’s perspective should concern the generation of particular outcome (the ‘generative perspective’). Thus, generative questions seek answers regarding how to bring about positively valanced phenomena (e.g. social cohesion). A second distinction in question framing concerns whether one is searching for evidence for a specific policy topic (the ‘informing-policy perspective’) or to inform general-purpose evidence search (the ‘evidence-search perspective’; see Table 1).

These two, new mutually exclusive ‘perspective’ types (generative, preventative) and (informing policy, evidence search) were applied to the full data set. Of all questions in the set, there appeared to be fewer preventative (15.3%) than generative (39.7%) questions. Also, there were approximately double the number of informing-policy (21%) compared to evidence-search (9.5%) questions. Given that the questions had been classified according to perspective, this enabled a further examination of patterns in perspective in relation to the topic and the style of the question concerned.

Overall, across all 7 topics, between 38.4 and 54% of questions sought answers that would inform generative questions (see Table 1); Defence and Security was associated with the highest proportion of this type. Far fewer questions across all topics were preventative in nature, ranging from 13.8 to 21.7%. Social Welfare (21.7%) and Health (21.2%) were the two topics with the highest proportion of preventative questions. Across topics, the proportion of evidence-search questions (range 7.4–25.2%) varied more than that of informing-policy questions (range 14.8–29.3%). The two topics that invited the most evidence-search responses were Technology (23.8%) and Climate and Environment (25.2%), and the most frequently generated policy-informing questions were for Defence and Security (29.3%).

Overall, across all 7 question styles, between 38.4 and 53.5% of questions were generative, with Causal (53.5%) and Procedural (51%) styles associated with the highest proportion. Preventative questions were less frequent occurring across the 7 question styles (range 14.5–24.5%), with Comparison (24.5), Causal (18.5%), and Procedural (18.3%) associated with the highest proportions. The proportion of evidence-search questions (range 6.1–11.7%) were overall lower than informing-policy questions (range 11–25.9%), where Example/Explanation (11.7%) styles had the highest proportion of evidence-search and Procedural (25.9) the highest informing-policy-perspective questions.

### Insights from structured interviews

The aim of exploring the qualitative data from the structured interviews was to ascertain what underlies the generation of questions by those working in public policy organizations. Given the way the structured interviews were set up—with 10 individuals who had taken up policy fellowships at two knowledge brokerages (CSaP, CAPE)—the analyses focused on their responses to two critical questions.

*What informed the way in which the questions were formulated?* The main basis on which the questions were originally formulated was to use them as a starting point to inform more detailed discussions, in order to understand a policy issue and how to address it (60%). In other words, most took an exploratory approach to information search. In contrast, 30% indicated that they generated their questions primarily for the purpose of attaining solutions-orientated responses (30%). While policy fellows from CSaP and CAPE were taking up policy fellowships individually, 70% of respondents reported that they had developed their questions by canvassing views from their teams or other academics so that the answers to them could be fed back to their respective teams for EBPM purposes. This suggests a greater level of collaborative effort in devising the policy questions than had been anticipated. 70% reported that they had taken time to make sure the questions were open (rather than closed), so as to extract maximal details that could be used for practical aspects of their day-to-day activities. This complements the findings from the quantitative analysis, as the majority of the policy questions were unbounded (~ 80%), and of the 7 different subordinate types most of the unbounded questions were procedural (~ 30%); these questions make requests for evidence that serve specifical practical issues (e.g. supporting improvements in measurement, interventions, or communication).

*What could be learned from the policy-academic exchanges overall?* The majority view of respondents was that the exchange with academics was a vehicle to step out of the day-to-day policymaking process to gain a ‘bigger picture’ of the policy issues, in order to prioritize next steps along the policy making journey (60%). The one-to-one exchanges were also seen as a means of challenging the way in which the senior policymakers themselves—as well as their organizations—go about interrogating a policy issue. As well as this,  the exchanges were seen as a way to expand the process of advice taking, including where to source the best advice by directly going to those that were involved in generating the evidence (50%). Knowledge brokerages were viewed by 50% as an opportunity to identify news ways of implementing policy. 30% use the exchange with academics to independently validate their understanding of a policy issue. In addition, 50% used the opportunity to meet with academics to help identify where they needed to revise or expand their understand of a policy issue through access to new sources of evidence recommended by the academics. The full set of findings from the structured interviews can be found in the supplementary materials (https://osf.io/h7duc).

## Discussion

The aim of the present mixed method study was to use the UK as a test case to examine if there are patterns in the topics that policymakers request evidence for, particularly if the same topic and the way the evidence for it is requested is made repeatedly over time, and across multiple government departments and governmental agencies. The quantitative component provided answers to these questions, and indeed revealed that there is considerable replication in effort, given how frequently the same style of requests for evidence were made for the same topic over time across multiple government departments and agencies. The qualitative component of this study points to potential ways to address this based on the insights from policymakers that align with recent empirical findings indicating the value of peer-to-peer exchanges between policymakers to support more cross governmental work.

The quantitative analysis revealed that, over a five-year period, across all five years there were 7 topics, the most common requests were for evidence informing policy issues around the Economy. The emerging topic that gained interest across time was Climate and Environment. For all 7 topics, the most common style of question didn’t restrict the types of answers invited (i.e. Unbounded). Of the specific question styles that fall under this broad category, Procedural questions were the most common, consistent with previous findings^8,9^. In addition, it is the most popular style of question overall—since it remained the most common across topics and across years.

The analysis of the questions also revealed new categories that captured the overall framing of the questions, where the most frequent invites answers to Generative (rather than Preventative) questions. This reflects greater interest in finding answers that could improve, change, or increase an outcome associated with a policy issue. While there might be an expectation that Defence and Security would, of all policy topics, would focus on Preventative questions, in fact Generative questions were most common for the Defence and Security topic and were typically styled as Causal or Procedural questions. By comparison, the Preventative perspective orientates questions towards preventing negative outcomes. Preventative questions were most common for the Social Welfare and Health topics. These were typically styled as Comparison-, Causal-, or Procedural-style questions. Another aspect of the overall framing of the questions concerns informing policy specifically or informing a general evidence-search approach. The latter was most common occurring overall, as well as most frequently over time in the Defence and Security topic and typically associated with Procedural style questions.

In the qualitative study structured interviews were conducted with individuals working in public policy organizations. The sample of interviewees were policy fellows that were spending time in knowledge brokerages interacting directly with researchers. The most consistent factor which underpins the way questions are generated is for the translation of evidence into potential solutions to policy problems^23,25,27–31^; when encountering evidence that researchers couched in technical terms, there were efforts in the one-to-ones to encourage researchers to reorientate their answers towards making more specific recommendations. Boaz and Oliver^17^ conducted interviews with 25 (out of over 250) participants in a UK-government knowledge-mobilization program, which provide valuable insights regarding the motivational factors that inform the generation of ARIs. Boaz and Oliver^17^ found that ARIs can be used across the academia-research boundary—for example, by “providing a mechanism for government departments to share their research interests with researchers”^17^ and as a mechanism for building connections (between government departments and between government and academia), sharing research interests with funders, and generated to achieve multiple goals.

The findings from the present qualitative work, as well as Boaz and Oliver^17^, complement each other and are insightful in further contextualizing what has been revealed from the quantitative data of the present study. The qualitative work reveals that questions reflect multiple interests; so, in the case of knowledge brokerages, policy professionals act as representatives of the teams and their evidence needs, just as ARIs represent the evidence needs of multiple teams with and across government departments. This is why many questions concern practical advice and invite researchers to refer to specific published findings, data sets, or work the researcher is conducting that the policy fellow or the team they belong to could follow up on. This also explains why the most common style of question that policy professionals ask (i.e. Procedural) is the most practical: it invites responses that articulate solutions-oriented outcomes (e.g. ways to improve measurement, communication to the public, or interventions). The qualitative findings also help to contextual why the framing of questions was commonly from a generative and informing-policy perspective, which steer responses towards *specific* policy issues and solutions. Finally, the iterative interactive exchanges between policymakers and researchers, as well policymakers to policymakers across different governmental departments, may be a way to avoid continuing replication of effort, because a more in depth focused interaction in which assumptions can be challenged, can break impasses where a policy problem continues to be addressed the same way using the same type of evidence.

We next turn to why policy questions help to further academia-policy interactions aimed at some form of EBPM. There is an overarching message we hope to promote in response concerns regarding the practical applications of the present work. Our proposal is that policy questions are a form of knowledge brokering in and of themselves, by which we mean a mechanism that facilitates the exchange of, and translation of, research and evidence to practitioners^31–37^. This can either occur directly in one-to-one meetings with academics via knowledge brokerages (e.g. CSaP, CAPE) or generally by accessing an academic community (e.g. ARIs).

As has been suggested^11,23,25–30^, among the many factors which hamper academic engagement in EBPM are the potential misconceptions around how evidence is interpreted^38–44^ and used in policy formation and policy decision-making^45–48^. Skepticism may be allayed to some degree if policy questions are viewed as a way to connect researchers to evidence users, by inviting this engagement as a starting point to promote more in-depth conversations. However, policy questions are clearly nuanced. Furthermore, each policy topic is multifaceted, so its solutions can come from different ways of interrogating—it through multiple academia-policy engagements. Also, a policy question posed to researchers doesn’t preclude other styles that could be further generated through co-productive activities. For instance, points of connection can be made. Researchers typically prefer to address questions of a Causal-Analytic style^9,12^, even if they are less frequently asked by policy professionals, those working in public policy organizations value the need to explains policy issues with a causal story^49,50^. Thus, the findings from this study help to illuminate the typical—as well as the range of—evidence needs, and these are starting points in what could be an ongoing knowledge brokering exchange that could help converge over solutions rather than diverge over misperceptions on what needs, and how evidence is used by policy.

Those working in public policy organizations can also benefit from the work presented in this study by taking the view that the questions they pose aid in the knowledge brokering process. Because the questions reflect the evidence needs of individuals, teams, or even entire departments, researchers’ contributions can be of benefit to a wide policy audience and across teams and government departments^17^. Not only can researchers use the pattern of findings in the topic and style of question to anticipate future evidence needs for EBPM, so can policy professionals. The way in which request for evidence and advice are styled varies, and some styles are more amenable to those that researchers are used to—such as Causal-Analytic style inquiries—than ones which make requests for practical recommendations^8–11^. In turn, policy professionals could complement the Procedural questions they favor with additional requests for evidence that account for the underlying mechanisms and causal factors that underpin policy issues or at least expect that researchers will be motivated in their exchanges to discuss their work on these terms. By understanding that there are important features of questions (e.g. perspective, topic, style) that signal important cues to their evidence needs, policy professionals are better armed at tailoring their request for evidence more precisely to engage with researchers. Given that the analyses presented here also reveal what style of questions are typical of a particular policy topic and over time, again by understanding these patterns, policy practitioners can use these insights to explore additional gaps in evidence needs not only with regards to their own teams and departments, but also in potential exchanges with other government departments^17^, as well with researchers.

### Limitations and future directions

The study has two important limitations. The first is that the data sets that the study was based on are drawn from the UK. While this restricts the generalizability of the trend analysis to other countries, TPQs is a general-purpose tool that can be applied to analyze any policy question from any governmental department or agency. For instance, as mentioned earlier, the EU and US have put into law EBPM. Policy questions will be a likely mechanism that articulates policymakers’ evidence needs to scientific experts when engaging with them early in the policy design process. Consequently, the present findings showing patterns in UK policymakers’ evidence needs can be used to conduct cross-country comparisons.

The second limitation is that it was not possible to analyze the kind of answers given by ‘evidence providers’ to policymakers’ questions. We cannot, therefore, determine whether the questions were sufficiently answered. One way to address this is to record the one-to-one discussions between researchers and policymakers. Independent coders can examine the transcripts to judge the alignment of the answer to the question. Furthermore, in the discussion’s participants could each give their assessment regarding the quality of each answer. Future work could also explore ways of gauging the factors that contribute to common beliefs about misalignment between the two communities by examining in real time the times of discussions that take place during roundtables and workshops. This would also be of value to knowledge brokerages that serve the needs of policy and science. We cannot improve the way policymakers are brokered ‘out’ to researchers, nor how researchers are brokered ‘in’ to policy, without knowing how to optimize the exchanges between the two.

A further development of TPQs would be to test for differences in the approach taken by policymakers (regarding questioning). Do policymakers take different approaches to questioning different scientific communities (e.g. climate scientists versus economists)? Furthermore, do policymakers from different advocacy coalitions^51^ approach questioning experts within their own coalition differently from those outside it?

Alternatively, are there differences regarding which experts knowledge brokerages deem most relevant given that they in turn act as gatekeepers. Perhaps experts answer questions designed to learn about a policy area (e.g. Bioethics) differently from those that examine the application of techniques that inform a specific policy (e.g. Bioengineering in the health domain). Perhaps questions concerning a given policy topic (e.g. Information Technology) focus on some disciplines (e.g. STEM), overlooking relevant insights from others (e.g. Social Sciences).

The main take away from raising these issues is that any future work that addresses them can further benefit scientific communities, knowledge brokerages, and those working in the policy domain so they can collectively work towards utilizing evidence effectively to support policy decisions.  

## Methods

### Ethics

Ethical approval for the data collected via interviews—was granted prior to conducting the study: Judge Business School, Departmental Ethics Review Group Approval, reference no, 22–24, 25-5-2022. Furthermore, the study complied with the Helsinki ethical principles for research involving human participants. Informed consent was obtained from all participants. The questions data sets were already publicly available and did not include any personal information (regarding age, gender, race, etc.). Furthermore, the analysis of these data sets did not concern group differences of any kind.

### Questions datasets

The only restriction, when compiling the set of questions, was the time period (2019 to 2023). The main rationale for focusing on this five-year period was to examine the trends in evidence requests prior to, and after, the COVID-19 pandemic, and that we could source data sets that would allow a comparison across them for a similar period. To be able to access data sets of this kind that we publicly available we turned to those that are regularly published on the UK government website: the ARI policy questions designed to invite evidence from academic (as well as non-academic) institutions to contribute to evidence needs from the UK Government. In addition, given that knowledge exchange and knowledge brokerages serve to support public policy organizations through policy fellowship schemes, this was also a potential way to access policy questions. Here policy fellows are invited to meet with academics to seek advice and to be better informed about research and evidence that could contribute to EBPM. The Centre for Science and Policy at the University of Cambridge is a knowledge brokerage which has compiled data sets of policy questions that policy fellows submit to the center to seek advice. In addition, the Capabilities in Academic Policy Engagement (CAPE; https://www.cape.ac.uk) is another example of a policy exchange network which also acts as a knowledge brokerage. The CAPE network is a partnership between five universities [Universities of Cambridge, Manchester, Northumbria and Nottingham] based at University College London). The CAPE network has also compiled data sets from the policy fellows that spend time meeting with academics in the network for advice and to access evidence that could be used for EBPM purposes. Given that CSaP and CAPE had data sets that they could make available for the purposes of analysis, we took the opportunity to use these along with the ARIs to conduct our analysis.

A total of 3930 questions were gathered from three sources (for details regarding the different government departments, agencies, and public bodies, and the aims in focusing on these sources see the supplementary materials: https://osf.io/h7duc). The sources were CSaP, University of Cambridge (*n* = 1670); CAPE network UK (*n* = 269), and the UK Government Office for Science’s annual published reports of ARIs along with corresponding research questions from government departments, agencies, and public bodies (*n* = 1991). Across all three datasets, the questions came from a total over 33 UK government departments, government agencies, and public bodies (for full details, see the supplementary materials: https://osf.io/h7duc).

### Qualitative dataset

While the sample for the structured interviews is small (*n* = 10), the insights from the responses are broadly informative of contextual factors that informed the generation and further development of questions. To this end, an opportunistic approach was taken to acquire the sample, where a request to take part in an in-depth interview was presented to those taking part in one-to-one policy fellowship schemes offered at CSaP and CAPE. 10 fellows responded to the request, and all were senior policy professionals from UK Government departments. All were then required to answer a series of five questions, which took approximately one hour. The five main questions were: (1) What does a valuable exchange look like?; (2) What do you think motivates an academic in this exchange?; (3) How honest can you be about some of the issues you face?; (4) What informed the way in which you formulated your questions?; (5) What is it that you can learn/have learnt from your exchanges? The responses were recorded and are presented as summaries in the supplementary materials (https://osf.io/h7duc), with the focus in the results section on responses to the last two questions.

### Coding questions dataset

The questions were coded in two ways, first by question style and second by topic. To classify the questions by style, the TPQs^8^ was used. This classification is specifically designed to identify question style (seeking information structured in specific ways) that typically come from policy—though it can, and has, been applied to classifying research questions posed by scientific researchers^9^. The taxonomy has two super-ordinate categories (Bounded, Unbounded) which identify questions by whether they are open-ended and invite the responder to provide answers that are detailed and expansive (Unbounded), or close-ended and invite the responder to give a specific and succinct response (Bounded). Within each super-ordinate category are sub-ordinate styles. The Unbounded (sub-ordinate) question styles are Procedural, Example/Explanation, Asserting Value Judgments, and Causal Analysis. Bounded (sub-ordinate) question styles are Verification, Comparison, and Forecasting. All 3930 questions were classified by two human coders. Coder 1 classified all 3930 questions, and five independent coders classified sub-sets of questions. In addition, as well as two human coders, an automated classification system was developed. In brief, common terms and phrases that were typical of each of the 7 sub-ordinate questions were used to generate an algorithm that sorted the questions, taking word length into account. The algorithm was insensitive to detecting any nuance in the phraseology of the questions that might help to further disambiguate the questions by style. Therefore, the classification was strict with respect to the presence or absence of key terms associated with each question style. Overall, the weakest agreement was between Coder 1 and Coder 5 (60%), and the strongest agreement was between Coder 1 and Coder 2 (75.60%); for a gauge of general accuracy 74.59% agreement was found between human coders for other comparable datasets^8^. (Though, this degree of agreement was the result of altering the categories of Graesser et al.’s taxonomy^18,19^ to account for overlaps^8^. The original degree of agreement—using the original categories—was 47.55%^8^.) When comparing human coders with the automated system, the weakest agreement was between Automatic coder and Coder 5 (55%) and strongest agreement was between Automatic coder and Coder 1 (70.10%). The full details regarding levels of agreement between coders and development of the automatic coding system are presented in the supplementary data file: https://osf.io/3eya6).

Having determined that the coding the questions by style, we use this to determine if we could meaningfully collapse across the three different data sets of questions for the main analyses conducted. The critical difference between the data sets from CSaP and CAPE and the ARIs were that the policy questions from the knowledge brokerages (CSaP, CAPE) were set by individuals from public policy organizations whereas the policy questions from ARIs are generated by teams of professionals. Despite this difference we compiled the questions into one single data set but provide details as to the source of each question (CSaP, CAPE, ARIs) along with the government department or agency that the questions came from to enable comparative analyses. Each data set was coded by super-ordinate and sub-ordinate categories to perform the comparison. CSaP data set: Bounded (18%), Unbounded (82%); Causal (19%), Comparison (4%), Example/Explanation (18%), Forecasting (3%), Procedural (35%), Value (10%), Verification (11%). CAPE data set: Bounded (19%), Unbounded (81%); Causal (15%), Comparison (4%), Example/Explanation (25%), Forecasting (5%), Procedural (27%), Value (15%), Verification (9%). ARIs data set: Bounded (13%), Unbounded (87%); Causal (18%), Comparison (3%), Example/Explanation (25%), Forecasting (5%), Procedural (29%), Value (15%), Verification (5%).

A check was performed to determine whether it was possible to collapse the questions from difference sources into a single data set to examine trends in topics and style of question. While it is not advisable to perform chi-squared analysis using percentages, exceptions to this can be made (see Richardson^52^). While we proceed with caution as to making strong inferences based on the findings from the analysis, the general indication is that, when examining the percentage of seven styles of questions in each of the three data sets, there did not seem to be a meaningful statistical difference when performing two forms of chi-squared analysis (*X*^*2*^ = 7.37 (*df* = 12, *N* = 300), *p* = .83; yates correction applied = 4.73 (*df* = 12, *N* = 300), *p* = .97).

The second scheme used to code the questions was topic—and algorithmic (for full details see the supplementary data file: https://osf.io/wp3ae). It was informed by previous approaches^8^. In short, the sets of questions were reviewed in turn and key terms were highlighted. The key terms were then sorted according to 8 core policy topics. From this, an algorithm was designed to detect all the key terms associated with the 8 topics, with a strong intention to only include key terms that did not overlap with any other topic. Even if multiple terms were identified in a single question, the score was converted to a binary code to enable fair comparisons across questions—otherwise, the comparisons could be unnecessarily skewed by few questions with multiple hits.

The criterion for including policy topics was set at a cut off at 330 questions (approximately 10% of all classified questions by topics) to enable meaningful analysis across all 5 years. Only one policy area did not contain enough questions to meet this criterion (Education [4.15%]). In addition, when applying the algorithm to the total dataset of questions (*n* = 3960) for topic detection, a portion of the questions did not generate hits for any of the 8 topics, (*n* = 670, 17.05%) and so were excluded from further analyses. This left a total of 3260 questions that were included in the final analysis. The questions uncoded by topic were still classified by style; the classification of these question style revealed that the least common style of question was Comparison (3.73%) and the most common style of question was Example/Explanation (28.50%). This is different to those questions that were in at least one of the 8 topics. This indicates some minor differences between uncoded questions by topic and those coded by topic (further detailed of the methods can be found in the supplementary materials (https://osf.io/h7duc), and analysis of this can be found in the supplementary data file: https://osf.io/wp3ae. In addition to the 8 main topics, the automatic classification system also identified the presence of terms that indicated a focus on how the policy topics ought to be addressed. Namely, whether the question’s perspective concerned evidence search [evidence/advice] or the use of evidence to make recommendations or inform policy [policy]. The second way in which the focus of the questions was signaled was with respect to whether the policy topic concerned developing policies, addressing issues that prevented an outcome from occurring, or generating a particular outcome. From this, it is possible to inform a more detailed analysis that nested these terms [evidence-search versus informing-policy perspective on some policy topic; preventative vs. generative perspective on a policy topic]. Therefore (see Table 1), 7 (out of a total of 8) policy topics met the inclusion criterion: Information Technology, Health, Economy, Climate and Environment, Social Welfare, Technology, Defence and Security.

## Electronic supplementary material

Below is the link to the electronic supplementary material.

Supplementary Material 1

Supplementary Material 2

Supplementary Material 3

## Data Availability

All data and supplementary materials are available at https://osf.io/bpxd7.

## References

[1] Kogan, M., Henkel, M. & Britain, G. *Government and Research: the Rothschild Experiment in a Government Department* (Heinemann Educational Books, 1983).

[2] Cairney, P. Evidence-based policymaking in Encyclopedia of European Union Public Policy. (eds (eds Tosun, J., Roberto, P. & Graziano) 138–147 (Cheltenham: Edward Elgar, UK, (2022).

[3] Oliver, K., Innvar, S., Lorenc, T., Woodman, J. & Thomas, J. A systematic review of barriers to and facilitators of the use of evidence by policymakers. *BMC Health Serv. Res.* **14**, 1–12 (2014). 24383766 10.1186/1472-6963-14-2 PMC3909454

[4] Pepper, S. *World Hypotheses: A Study in Evidence* (University of California Press, 1942).

[5] Davies, P. T. Is Evidence-Based Government Possible? Jerry Lee Lecture, presented at the 4th annual Campbell Collaboration Colloquium. Washington DC, USA. *DC*, (2004). http://webarchive.nationalarchives.gov.uk/20091013084422/http://www.nationalschool.gov.uk/policyhub/downloads/JerryLeeLecture1202041.pdf. *February*.

[6] De Marchi, G., Lucertini, G. & Tsoukiàs, A. From evidence-based policy making to policy analytics. *Ann. Oper. Res.* **236** (1), 15–38 (2016).

[7] Oliver, K., Lorenc, T. & Innvær, S. New directions in evidence-based policy research: a critical analysis of the literature. *Health Res. Policy Syst.* **12**, 1–11 (2014). 25023520 10.1186/1478-4505-12-34 PMC4107868

[8] Osman, M. & Cosstick, N. Finding patterns in policy questions. *Sci. Rep.* **12**, 1 (2022a). 36418853 10.1038/s41598-022-21830-z PMC9684423

[9] Osman, M. & Cosstick, N. Do policy questions match up with research questions? No.1. *Centre for Science and Policy, University of Cambridge Working Paper series.* (2022). https://osf.io/t6a2y

[10] Osman, M. & Cosstick, N. How do different groups judge the quality of research questions which inform Evidence-Based policymaking?? *J. Edu Psyc Res.* **6**, 01–15 (2024).

[11] Trafimow, D. & Osman, M. Barriers to converting applied social psychology to bettering the human condition. *BASP* **44**, 1–11 (2022).

[12] Trafimow, D., Cosstick, N. & Osman, M. Problem testing (and beyond) in the messy world of public policy. *Sci. J. Rev.* **4**, ID000586 (2024).

[13] Cairney, P. The UK government’s COVID-19 policy: what does guided by the science mean in practice? *Front. Polit Sci.* **3**, 624068 (2021).

[14] John, S. Should science lead? *Philosophers’ Magazine*. **90**, 58–63 (2020).

[15] John, S. The two virtues of science. *Spontaneous Generations: J. History Philos. Sci.* **10**, 47–53 (2022).

[16] Oliver, K., Boaz, A. & Cuccato, G. Areas of research interest: Joining the dots between government and research at last? *F1000Research* 11 (2022). 10.12688/f1000research.127542.2 PMC12924004 41727908

[17] Boaz, A. & Oliver, K. How well do the UK government’s ‘areas of research interest’ work as boundary objects to facilitate the use of research in policymaking? *Policy Polit*. **51**, 314–333 (2023).

[18] Graesser, A. C., Person, N. & Huber, J. Mechanisms that generate questions. In T. W. Lauer, E. Peacock, & A. C. Graesser (Eds.), *Questions and Information Systems* 167–187Lawrence Erlbaum Associates, (1992).

[19] Graesser, A. C., McMahen, C. L. & Johnson, B. K. Question asking and answering. In (ed Gernsbacher, M. A.) Handbook of Psycholinguistics, 517–538. (Academic, (1994).

[20] Miyake, N. & Norman, D. A. To ask a question, one must know enough to know what is not known. *J. Verb Learn. Verb Behav.* **18**, 357–364 (1979).

[21] Pomerantz, J. A linguistic analysis of question taxonomies. *J. Am. Soc. Inf. Sci. Tec.* **56**, 715–728 (2005).

[22] Gollust, S. E. et al. Mutual distrust: perspectives from researchers and policy makers on the research to policy gap in 2013 and recommendations for the future. *INQUIRY: Inq. (INQ)*. **54**, 0046958017705465 (2017). 10.1177/0046958017705465 PMC5798731 28452251

[23] Walker, L. A., Lawrence, N. S., Chambers, C. D., Wood, M., Barnett, J., Durrant, H.,… Kythreotis, A. P. Supporting evidence-informed policy and scrutiny: A consultation of UK research professionals. PloS one, 14(3), e0214136 (2019). 10.1371/journal.pone.0214136 PMC6435130 30913236

[24] Oliver, K. et al. Assessing the Overlap between UK Government Knowledge Priorities and Funder Portfolios. *Available at SSRN 4998285*.

[25] Bozeman, B., Bretschneider, S., Lindsay, S., Nelson, J. P. & Didier, N. Reports of practitioners’ use of public affairs faculty published research. *Stud. High. Educ.* **48**, 719–732 (2023).

[26] Reichmann, S. & Wieser, B. Open science at the science–policy interface: bringing in the evidence? *Health Res. Policy Syst.* **20**, 70 (2022). 35725491 10.1186/s12961-022-00867-6 PMC9208144

[27] Osman, M. Misdiagnosing the problem of why behavioural change interventions fail. *Behav Brain Sci*, 46 (2023). 10.1017/S0140525X23001085 37646256

[28] Walsh, J. C., Dicks, L. V., Raymond, C. M. & Sutherland, W. J. A typology of barriers and enablers of scientific evidence use in conservation practice. *JEM* **250**, 109481 (2019). 10.1016/j.jenvman.2019.109481 31518795

[29] Bogenschneider, K. & Corbett, T. *Evidence-based Policymaking: Envisioning a New Era of Theory, Research, and Practice* (Routledge, 2021).

[30] Parkhurst, J. *The Politics of Evidence: from Evidence-based Policy To the Good Governance of Evidence* (Taylor & Francis, 2017).

[31] Gluckman, P. D., Bardsley, A. & Kaiser, M. Brokerage at the science-policy interface: from conceptual framework to practical guidance. *Humanit. Soc. Sci. Commun.* **8**, 84 (2021).

[32] Pielke, R. *The Honest Broker* (Cambridge University Press, 2007).

[33] Bandola-Gill, J. Knowledge brokering repertoires: academic practices at science-policy interfaces as an epistemological bricolage. *Minerva* **61**, 71–92 (2023).

[34] Bandola-Gill, J. & Lyall, C. Knowledge brokers and policy advice in policy formulation. In Handbook of Policy Formulation (249–264) (eds Howlett, M. & Mukherjee) ( and I) (Edward Elgar Publishing, (2017).

[35] MacKillop, E., Quarmby, S. & Downe, J. Does knowledge brokering facilitate evidence-based policy? A review of existing knowledge and an agenda for future research. *Policy Politics*. **48**, 335–353 (2020).

[36] Bednarek, A. T., Miyamoto, B., Corbett, K., Hudson, C., Scarrow, G., Brass, M., …Kolavalli, C. How and why funders support engaged research. PNAS, 122, e2400931121 (2025). 10.1073/pnas.2400931121 PMC11725928 39793032

[37] Shaxson, L., Hood, R., Boaz, A. & Head, B. Knowledge brokering inside the policy making process: an analysis of evidence use inside a UK government department. *Evid. Policy*. **1**, 1–20 (2024). 10.1332/17442648Y2024D000000028 40131806

[38] MacKillop, E. & Downe, J. What counts as evidence for policy? An analysis of policy actors’ perceptions. *Public. Adm. Rev.* **83**, 1037–1050 (2022).

[39] Mullen, E. J. Reconsidering the ‘idea’ of evidence in evidence-based policy and practice. *Eur. J. Soc. Work*. **19**, 310–335 (2016).

[40] Murad, M. H., Asi, N., Alsawas, M. & Alahdab, F. New evidence pyramid. *BMJ Evid. Based Med.* **21**, 125–127 (2016). 10.1136/ebmed-2016-110401 PMC4975798 27339128

[41] Nutley, S., Walter, I. & Davies, H. T. From knowing to doing: A framework for Understanding the evidence-into-practice agenda. *Evaluation* **9**, 125–148 (2003).

[42] Cairney, P. & Kwiatkowski, R. How to communicate effectively with policymakers: combine insights from psychology and policy studies. *Palgrave Commun.* **3**, 37 (2017).

[43] Ish-Shalom, P. Theorizing politics, politicizing theory, and the responsibility that runs between. *Perspect. Politics*. **7**, 303–316 (2009).

[44] Hetherington, E. D. & Phillips, A. A. A scientist’s guide for engaging in policy in the united States. *Front. Mar. Sci.* **7**, 409 (2020).

[45] Bednarek, A. T., Wyborn, C., Cvitanovic, C., Meyer, R. Colvin, R. M, Addison, P. F.,… Leith, P. Boundary spanning at the science-policy interface: The practitioners’perspectives. Sustain. Sci. 13, 1175–1183 (2018). 10.1007/s11625-018-0550-9 PMC6086300 30147800

[46] Cairney, P. *The Politics of Evidence-Based Policy Making* (Springer, 2016).

[47] Howlett, M., McConnell, A. & Perl, A. Moving policy theory forward: connecting multiple stream and advocacy coalition frameworks to policy cycle models of analysis. *Aust J. Public. Adm.* **76**, 65–79 (2017).

[48] Pluchinotta, I., Daniell, K. A. & Tsoukiàs, A. Supporting decision-making within the policy cycle: techniques and tools. In (ed Howard, M.) The Routledge Handbook of Policy Tools 235–244 (Routledge, (2022).

[49] Stone, D. A. Causal stories and the formation of policy agendas. *Political Sci. Q.* **104**, 281–300 (1989).

[50] Jones, M. D., Smith-Walter, A., McBeth, M. K. & Shanahan, E. A. The narrative policy framework. In Theories of the Policy Process (161–195). (Ed. (2023). Weible C. M.) (Routledge, Taylor & Francis.

[51] Sabatier, P. A. & ‘Knowledge, P. O. Learning, and policy change: an advocacy coalition framework’. *Sci. Commun.* **8**, 649–692 (1987).

[52] Richardson, J. T. The analysis of 2 × 2 contingency tables—Yet again. *Stat. Med.* **30**, 890–890 (2011). 21432882 10.1002/sim.4116

