# Supplementary material for: Understanding UK policymakers’ evidence needs through policy questions
Source: Sci Rep. 2025 Jul 2;15:22484. doi: 10.1038/s41598-025-05911-3 (PMC12215802; doi:10.1038/s41598-025-05911-3)
Supplement: Supplementary file 3 — Supplementary Material 3 [file 41598_2025_5911_MOESM3_ESM.pdf]

Understanding UK policymakers evidence needs through policy questions

Magda Osman[1,2]* & Nick Cosstick[1]

1 Judge Business School, University of Cambridge, Trumpington Street, Cambridge, CB2 1AG UK

2 Leeds Business School, University of Leeds, Maurice Keyworth Building, Woodhouse, Leeds LS2 9JT

*m.osman@jbs.cam.ac.uk

Organisation of this document

The project involved two sources of data, the first is datasets of questions posed by policy professionals from the UK Civil Service and compiling them from several available sources into one complete set gathered over the past 5 years (2019 to 2023). The second set of data was from 12 structured interviews that were conducted from November 2022 to May 2023.

The interviews were based on presenting senior policy makers that had contributed to the questions included in the primary dataset, and involved interviewing the policy makers as to the basis on which the questions were developed and what the intended use of the answers from academics would be.

This document is divided into two, with the first outlining the process of gathering the data for the Questions data sets, and the coding frame that was used, and the second section presents the details regarding the interview questions, and a summary of the responses to them[1].

[1] To preserve the anonymity of the respondents, the responses to the questions were summarised so that a complete transcript of the interviews is not available given that the responses contained details that would directly identify the respondents and the organisations they belong to.

Questions Data sets

There were three sources of data that were used to compile the complete data set of research questions posed by policy professionals from the UK Civil Service.

Centre for Science and Policy: https://www.csap.cam.ac.uk/. From the period of 01-01-2019 to 01-05-2023 there were a total of **1670** questions (see Supplementary Table 1) from policy professionals taking up a policy fellowships at Centre of Science and Policy (CSAP), University of Cambridge, UK. This data set also include questions from policy fellows that have applied to the fellowship scheme for which the fellowship is currently being processed, so these are the most up to date questions that are included here and in the entire data set of questions compiled.

The fellowship involves policy professionals presenting a set of questions that they will use to base their one-to-one discussions with academics. The academics have different subject expertise, and they are invited to respond to the questions posed by meeting for hourly sessions with the policy professionals to either directly answer the questions, or more generally talk around the policy area the policy fellow is working on. The academics that volunteer to talk with policy fellows are not exclusively from the University of Cambridge, though the majority are often local to the university, however, the network of academics on the system are from universities across the UK.

Capabilities in Academic Policy Engagement (CAPE): https://www.cape.ac.uk/. From the period 01-01-2021 to 01-05-2023 there were a total of **269** questions from policy professionals taking up fellowships at CAPE (see Supplementary Table 1). The CAPE policy fellowship scheme is the same as the CSaP policy fellowship scheme but in this case includes a specific set of academic institutions from which policy fellows can meet with. The academics are from 5 academic institutions: University of Cambridge, University College London, University of Manchester University of Northumbria and University of Nottingham.

Areas of Research Interest (ARIs): https://www.gov.uk/government/collections/areas-of-research-interest. From the period 01-01-2019 to 01-05-2023 there were a total of **1991** questions from policy professionals taken from published reports on the UK Government Office for Science website. The UK Government publishes ARIs from many different government departments (N = 19), government agencies, and public bodies (N = 4). The aim here is to indicate to academics and research institutes the specific themes that various government departments, agencies and public bodies are interested in addressing through the

use of evidence, and provides a public record of their evidence needs. The style of these reports varies from department to department, with some focusing on identifying specific research themes and only identifying those, whereas other ARIs include a comprehensive list of specific research questions that they are currently interested in. Supplementary Table 1 provides a breakdown of the different UK Government departments, Government Agencies, and Public Bodies that have published questions that are included in the dataset.

Supplementary Table 1. UK Government Departments, Government Agencies and Public Bodies from which research questions were gathered for the Questions dataset.

Examples of Government Depts, Agencies and Public Bodies included in the data sets of Qs.[2]	CAPE	CSaP	ARIs	No. of ARIs Qs
Cabinet Office	2021-2023	2019-2022	2019	93
Counter-Terrorism Policing HQ		2021-2023		
Department for Business, Energy & Industrial Strategy[3]	2021-2023	2019 - 2023	2020	272
Department for Digital, Culture, Media and Sport		2019-2022		
Department for Education		2019-2022		
Department for Environment Food and Rural Affairs	2021-2022	2019-2022	2021	166
Department for International Trade		2019-2022	2020	187
Department for Levelling Up, Housing and Communities	2021-2023	2019-2022	2022	239
Department for Transport		2019-2022	2021	324
Department for Work and Pensions		2019-2021		
Department of Health & Social Care	2022-2023	2019-2023		
Financial Services Group, HM Treasury		2022		
Food Standards Agency		2020	2020	11
Foreign, Commonwealth & Development Office	2021-2022	2019-2023	2020	40
Government Office for Science		2019-2022		
Health and Safety Executive			2021	82

[2] Not all UK Government Departments, Government Agencies and Public Bodies are listed here because some of the policy fellows taking up their fellowships at CSaP were high ranking public officials that did not want to make known the organisations they were representing while taking up their fellowship. Nonetheless, the list provided in Table 1 provides a comprehensive list of organisations, and gives an accurate record of the dates from which the questions were posed to the academic community.

[3] Some of the names of government departments have changed. For instance, Department for Business, Energy and Industrial Strategy no longer exists, and is now divided into three departments: Department for Business and Trade, Department for Energy Security and Net Zero, and Department for Science, Innovation and Technology. Also, the Department for Digital, Culture, Media and Sport no longer exists, and is now Department for Culture, Media and Sport, and Department for Science, Innovation and Technology.

HM Courts & Tribunals Service		2022		
HM Revenue & Customs		2020-2022		
Home Office		2019-2021		
International Trade Committee			2021	39
Ministry of Defence		2019-2023		
Ministry of Justice		2019-2022	2020	166
National Archives			2020	20
Number 10 Policy Unit	2021-2022	2021		
Office of National Statistics		2019		
Ofgem		2022		
Parliamentary Office of Science and Technology			2020	352
UK National Audit Office		2019		
UK Space Agency	2021-2022			
UK Statistics Authority		2020		
Valuation Office Agency		2022		
Total	269	1670		1991

Preparing the complete data set.

The full data set of 3930 questions can be found here (https://osf.io/3eya6). The file is split according to the different years and different sources that the questions were taken from. The file includes the year and the location (e.g. 2021 CSaP) and then the question itself. There are no identifiers as to the UK Government dept, or the name of the policy fellow, so as to preserve the anonymity of the individuals taking part in the fellowships. Moreover, the main focus of the analysis was to look at overall trends in themes, irrespective of the particular organisations that the policy professionals belong to.

The other critical details included in the complete data set were the questions classified by type according to the Osman and Cosstick (2022a) Policy Questions Taxonomy[4] (see Supplementary Table 2 for full details). The classification system developed by Osman and Cosstick (2022a, 2022b[5]) was applied to each question. The process of classification was

[4] Osman, M., & Cosstick, N. (2022a). Finding Patterns in Policy Questions. *Scientific Reports*, *12*(1), Article 1. https://doi.org/10.1038/s41598-022-21830-z

[5] Osman, M., & Cosstick, N. (2022b). Do policy questions match up with research questions? No.1. *Centre for Science and Policy, University of Cambridge Working Paper series. https://osf.io/t6a2y*

stringent in the following two ways, first two independent coders classified each question for a given data set, and then the same sets of questions were submitted to an algorithm developed as an Excel Macro that automatically classified the questions according to features that could assign each question into one of 7 types (see Supplementary Table 2). A total of 5 coders were used, to ensure that there were two human coders classifying each question, one of the five coders was the consistent for all coding of all 3930 questions.

The value of this was to ensure a high level of consistency throughout, though the addition of another human coder and the automated system were designed as sense checks to ensure consistency as well as to identify human errors that could be made, as well as resolving ambiguity in the classification of some questions; some questions were nested such that they contained multiple questions in one, and so this added some ambiguity as to which was the most salient and critical question that was being asked.

Supplementary Table 2. Osman and Cosstick (2022) Taxonomy of Policy questions: Function of expected answers

Super-ordinate category	Sub-ordinate category	Abstract specification	Example
Bounded Answers	Verification/ Qualification	Is it the case that X is here? Did X event occur? Are Xs more inclined towards y? Is X a viable version of Y?	Do groups generally make better decisions than individuals? What is the justifications for using groups to make decisions?
	Comparison	What are the strengths and weaknesses of X? What are the costs and benefits of implementing X?	What are the costs and benefits of groups over individuals?
	Forecasting	Which areas would you foresee improving in the next 10 years? How likely is it that X will be popular in the future?	How will group decision-making shape action in the next 10 years?
Unbounded Answers	Example/Explanation	Which X is more like Y? What would be a case where Y is like X? How does X work?	Can you illustrate situations where groups make better decisions? How does group decision-making work?
	Casual Analysis (antecedents or consequences)	What are the barriers that will prevent X from occurring? What are the effects of X if it is implemented now?	What happens if groups end up making the wrong decisions? What brings about the need to use groups in situations of adversity?
	Instrumental /procedural/Enablement	How can we use X to make Y better? What would need to be incorporated to ensure that X is produced? In what way can we measure X so that it can later be used to support y?	What strategies can organisation X implement for group decision-making to occur? What are the methods and strategies by which individuals can be encouraged to work in groups?

	Explaining/asserting Value judgments	How should the infrastructure available be used to produce x? How should X respond to y?	Why do you think groups are the best way to make decisions?

Automated Classification system: As well as using human coders, the aim of developing an automated classification system was to facilitate the speed by which questions of the type included in this questions data set could be efficiently classified in the future. The main reason for developing the automated system was also to help in situations where there was disagreement between human coders as to the classification of questions, as well as to determine which of the two was most closely aligned with a system that was unbiased in classifying questions.

To develop an automated system, the process was devised to take into account two features, the first of which was to find the most distinctive words or phrases most commonly associated with a question type, and the word length, so that the number of "hits" of terms associated with a given questions time was proportional to the number of total words included in the question itself. These were the two critical properties that were used to inform the automated system, which was immune to any nuanced aspects of a question, and in a blunt and simple way classified questions according to "hits" by specific words/phrases and taking into account question length.

To determine common words/phrases associated with each of the 7 question types, previously classified questions were used (Osman & Cosstick, 2022a, 2022b). The data sets of questions generated by policy were drawn from the same sources (e.g. CSaP and ARIs) and so where comparable to the datasets included in the present study. There were approximately 4,500 questions that had been classified by hand by at least two human coders. The coder that had the highest level of agreement across all human coders used was used as the master coded data set. From this, the questions that had been classified were split into individual files for further analysis. The data set corresponding to each type of question was then used to first identify the most common terms and phrases, this was done by simply looking at word/phrase frequencies. This was further supplemented by reading through a sample of questions selected at random to identify other words and phrases that had not been flagged by the frequency counts. This was repeated three times over, so that the detection of critical words/phrases was both representative of the question type, as well as included unique features of the question type that could help in

the classification process. This process was implemented for each of the 7 sub-ordinate question types (see Supplementary Table 2).

From this, a complete list of words/phrases was compiled for each of the 7 sub-ordinate question types. Then from this, the words/phrases that were compiled were cross checked so that where possible only unique words/phrases were identified and where there were repetitions of words/phrases appeared across the 7 sub-ordinate questions, they were removed. Again, the aim here was to refine the process of classification so that only "unique hits" would occur for a question, to avoid, where possible a single question being classified under more than one type of question. However, it was unavoidable that some questions were likely to be classified under more than one type of question for the reason that the phrasing was ambiguous, nuanced, and also was nested, such that there were multiple questions contained in one overarching question posed.

The number of possible hits given the length of the question was refined because the number of unique hits varied by type of question, for instance, questions that fell under the category of forecasting had words/phrases that were more distinctive (e.g. forecasting, anticipate, future) that did not appear frequently in other question types, as so a single hit for a short question that was 6 words long was sufficient. For other question types such as Causal Analysis distinctive words/phrases were harder to find because there was cross over with Example/Explanation type questions which also subsumed similar words/terms as Causal Analytic question types. To resolve this, the proportion of "unique hits" was weighted in such a way that core terms for causal analytic (e.g. consequence, effect, affect, causal, mechanism) had to appear more often in a question by a given word length, than for Example/Explanation type questions, were the weighting was implemented to reflect words/phrases that were more commonly associated with this type of question.

Once a prototype automated system was devised, it was submitted to the 4500 human coded bank of questions to determine the accuracy rate. The first prototype was 62% accurate. Adjustments were made to the weighting – which was based on the number of possible hits of key words/terms by the number of total words in a question, so that the accuracy rate could be increased. By doing this, the accuracy rate increased to approximately 68% which was comparable to the agreement between the two human coders. It is worth nothing that the accuracy rate may still be deemed relatively low, where 80-90% accuracy should be the aim,

however, as mentioned before, some questions are phrased in such a way that they are highly ambiguous, they also fall under multiple types, and in some cases could be interpreted as statements rather than questions, as a result the automated system faces the same issues as human coders face. In addition, where there was disagreement between human coders as to the classification of questions, the disagreement was predominately for questions that fell under the same super-ordinate category, and where ambiguity was expected – that is questions could often be classified as example/explanation or causal analytic, or causal analytic and procedural – which also suggests that there are root concepts that all three types of question types potentially draw on. Supplementary Table 3 summarises the percentage agreement between human coders for each of the questions classified for the current 3930 data set of questions spanning 2019-2023, and also includes the percentage agreement between the automated system and each human coder (https://osf.io/3eya6).

As is indicated in Supplementary Table 3 there is variability in the level of agreement by coder. There was a total of five human coders, with Coders A and C, and Coders A and E with the closest agreement, and Coders A and B with the least agreement. Also, while the automated coding system did not reach the same high level of agreement between those of the human coders, when performing well, it was within the range of agreement of human coders (e.g. ~65%).

Also indicated Supplementary Table 3 is how the automatic coding system performed. The way in which it applied the classification process differed from the human coders given that they were required to classify each question, and to assign it to one of the 7 question types. The automatic coding system had greater flexibility. It could classify questions into a single unique question type (single), or the question received multiple hits, and so appeared in more than one sub-ordinate category (multiple), or it did not get assigned to any category (uncoded). On average, about 34% of the questions in the different data sets were classified into a single category, approximately 53% were classified into multiple categories, and approximately 12% were uncoded. Given that more questions were classified into more than question type, this increased the possibility of a hit with the classification of the question by a human coder. The automatic coding system was quite basic and did not apply any nuance to the way it identified terms, for instance it could have identified words appearing a particular phrase rather than simply key terms or a simple phrase. Nonetheless, given this, the agreement between human coders and the automated classifier system was reasonable.

Supplementary Table 3. Percentage agreement of classification of questions by source and by coder.

Human Coders	Data Source	Human Coder 1 and Human Coder 1 % agreement	Human Coder 1 and Automatic Coder % agreement	Human Coder 2 and Automatic Coder % agreement	Automatic coder[6] - % unclassified (U), % single (S), % multiple (M)
Coder A, Coder B	CSaP 2023 (n = 211)	60	63.92	57.73	U = 8.06, S = 39.81 M = 52.12
Coder A, Coder C	CSaP 2021-2022 (n = 582)	75.60	65.32	64.11	U = 14.78, S = 33.33 M = 51.89
Coder A, Coder E	CSaP in progress Q 2021-2022 (n = 186)	74.19	70.06	69.46	U = 10.22, S = 36.02 M = 53.76
Coder A, Coder D	CSaP 2019-2020 (n = 514)	68.87	68.78	66.97	U = 14.01 S = 36.77 M = 49.22
Coder A, Coder E	CSaP in progress Q 2019-2020 (n = 177)	71.18	57.96	67.52	U = 11.30 S = 30.51 M = 58.19
Coder A, Coder B	CAPE 2021-2022 (n = 269)	61.13	66.82	55	U = 18.22 S = 31.60 M = 50.19
Coder A, Coder C	ARIs 2021-2022 (n = 1141)	71.29	63.66	61.27	U = 12.09 S = 33.30 M = 54.60
Coder A, Coder C	ARIs 2019-2020 (n = 850)	72.83	63.61	66.80	U = 12.35 S = 32.82 M = 54.82

[6] The % presented in this column indicate the number of questions that were classified according to more than one question type (which also increases the likelihood of a "hit" with a human coder's classification of the questions), and the % of questions that received a unique single classification, and the % that were uncoded by the automated classification system.

Thematic analysis

The coding of the questions by theme was conducted in a similar way to the automated system of classifying the questions by type according to the Taxonomy. In addition, an automated system had already been developed and used on previous data sets (Osman & Cosstick, 2022a, 2022b). Questions that had been compiled from the same sources that had been previously analysed (Osman & Cosstick, 2022a, 2022b) were first used to form the key terms, first by looking at work frequency, and the developing categories of themes along the lines that are often common as core policy themes (see Supplementary Table 4). The terms were moderately updated (see https://osf.io/wp3ae) to reflect the fact that terms referring to the same phenomenon change over time and also new terms enter into the vernacular (e.g. online instead of internet, AI as shorthand for Artificial Intelligence etc..).

To include the most up to date terms under the same 8 policy themes, the 3930 questions were compiled into file, and common terms and phrases were identified, the same data set was then submitted to word clouds to also help identified key terms that were missed. In addition, while classifying the questions by type, coders were also asked to note unique terms or phrases that indicated the focus of the theme of the question, so that these could also be included in the automated coding system. The automated coding system was a macro generated in Excel (see "RAW" tab in https://osf.io/wp3ae) which simply assigned a value to the number of times a key term or phrase appeared in a question under a give theme. Then the number of "unique hits" were converted in to a binary code (1 = hit, 0 = miss) so that percentages could be calculated, otherwise the number of total hits could distort the analysis (e.g. of all 3930 questions there might only be 10 that included terms associated with Defence and Security, but if the length of those 10 questions is long, and the number of hits could be 200, this would give a false impression of the distribution of questions by theme).

As well as the 8 main policy themes, four other themes were included, two fell under the broad category of approach to address the policy issue (i.e. generative vs. preventative) and focus in examining the policy issue by considering the perspective of institutions and mechanisms for policy making, or else from the perspective of evidence generation (i.e. policy vs. evidence). The aim here was to add further dimensions for considering the policy themes with respect to how they might be approached, namely through the prevention of an outcome, or through introducing processes that could generate a particular outcome. The themes were emergent from the way in which the questions were reviewed and coded, and so to reflect the fact that these dimensions were common across all question examined, the automated coding system

was adapted to identify words/phrases that were associated with them. Moreover, this enabled a further detailed analysis of the themes of the questions (see Supplementary Table 4 for details).

Supplementary Table 4. Main themes and associated terms used to code the policy questions by theme

Main Themes	Associated terms	No. of associated terms
Information Technologies	“artificial intelligence” “digital” “smart” “ICT” “Big Data” “machine learning” “algorithms” “software” “cyber” “cryptocurrencies” “quantum computing” “data privacy” “blockchain” “internet of things”	14
Economics & Finance	“economics” “economy” “econometrics” “taxes” “finance” “financial” “incentives” “supply chains” “labour markets” “assets” “markets”	11
Education	“education” “educate” “schools” “schooling” “universities” “university” “teachers” “teach” “educators” “pupils” “students” “academia” “academics” “curriculum”	14
Climate and Environment	“environment” “climate change” “green energy” “sustainability” “sustainable” “decarbonisation” “pollution” “pollutants” “weather” “renewable energy” “carbon footprint” “anthropogenic” “fossil fuels”	13
Defense and Security	“defence” “defense” “security” “threat” “combat” “deterrence” “decommission” “secure” “attack” “attacks” “terror” “terrorist” “terrorism” “extremism” “halt” “counter”	17
Health	“health” “medical” “medicine” “patients” “doctors” “medics” “diseases” “wellbeing” “mental health” “diagnosis” “obesity” “diet” “treatment” “diagnostics” “illness” “virus” “lifestyles”	17
Social Welfare	“Welfare” “wage” “earnings” “food insecurity” “equality” “quality of life” “disparity” “discrimination” “bias” “injustice” “human rights” “race” “social mobility” “public trust” “pay” “disability” “elderly”	17
Technology	“technology” “engineering” “engineer” “technological innovations” “industrial” “manufacturing” “construction” “infrastructure” “research and development”	10
Generative	“generate” “increase” “enhance” “maximise” “growth” “improve” “facilitate” “encourage” “stimulate” “harmonize” “evolve” “synergize” “surmount” “develop” “accomplish”	15
Preventative	“prevent” “minimize” “mitigate” “attenuate” “discourage” “lower” “constrain” “decrease” “avoid” “curb” “control” “shrink” “interrupt” “block” “constrain”	15
Policy	“policy instrument” “policy outcome” “public impact” “policy process” “policy design” “policy makers” “national policy” “global policy” “policy delivery”	9
Evidence	“evidence” “advice” “research” “expertise”	4

Structured interviews

The main rationale for conducting interviews was to provide an additional dimension for understanding the basis on which the questions devised by policy makers and what the expectations are with respect to engagement with them, and what might be done with the responses.

The details present here outline the foundations for why the interview questions were structured in the way that they were. The main objective of structured interviews is to gather data that can be used to facilitate effective co-production at the early stages of exchanges between policy professionals and academics.

Through a series of 10 one-hourly interview sessions, each of the 10 policy fellows were in dialogue with one of the researchers of this project. The structured interviews were conducted online, and followed the same procedure with the same sets of questions presented in the same order.

Phases of one-hourly structured interviews:

> Constructive-deconstruction element 1 [intentions-self]: The starting point is to work backwards by encouraging the policy fellows to consider what the anticipated outcomes of the exchanges with academics are and how realistic they are (e.g. What would a valuable exchange be for you? What might be a less valuable exchange?).
>
> Constructive-deconstruction element 2 [intentions-other]: The next stage is to also consider what the interests are of those in the exchange. Here the policy fellow will be invited to consider what the interests and motivations of the academic might be in an anticipated exchange (e.g. what do you think an academic might what to know from you? What kind of things do you think an academic might be interested in telling you about?).
>
> Deconstructive element 1 [challenges]: The aim here is to help expose what the specific exchanges with academics could be that present possible challenges (e.g. Is there an opportunity to discuss with academics the challenges you face around the policy issue you are interested in? How honest can you be about to the barriers and practicalities to addressing the policy issue you are considering during your fellowship?)
>
> Constructive element 1 [opportunities]: The aim here is to expose what the policy fellow is open to in an exchange with an academic, and how that can inform the type of exchanges they will have (e.g. Are you using the exchanges to gain advice on something in particular? Could you use the fellowship as a way to think about different approaches to understanding your policy issue?)
>
> Deconstructive element 2 [goals]: The aim here is to encourage the policy fellow to consider the underlying views and assumptions that underpin the way they are thinking about the policy issue (e.g. what is the starting point of the policy issue

and what assumptions does it depend on? How did this influence the way the policy questions were formed that you proposed as part of your fellowship?

Constructive element 2 [goals]: The final component is to examine whether there are ways in which the types of inquiries that policy fellows are considering as part of their fellowship could be adapted (e.g. What is it that you really need to know? What are you most interested in finding out and learning from?).

Motivations behind this approach

The main motivations behind this approach were to encourage a personal critical reflection of what the policy fellowship is being used for, and in particular the questions that drive the entire fellowship. There are many reasons that a policy professional will take up a fellowship which combine personal interests with interests of the organisation they belong to. Both types of interests need not be in conflict with each other, but they may lead be explicit considered, and so the aim of this pilot scheme is to given policy fellows an opportunity, in a structured manner, to think through what is of value.

Often the priorities that govern the exchanges are not explicitly articulated, and where they are they may be typically presented in practical terms – e.g. I need to understand what the existing evidence base is to address policy issue x, or I need to know what the best approaches are to innovating the way policy issue x is addressed[7] [8].

Recruitment for potential interviewees was based on sending out requests to the CAPE network of policy fellows and to CSaP policy fellows during the period of 01-10-2022 to 01-05-2023 to take part in an hour long interview. The interview was pitched also as a type of self-reflection session that could be used to encourage those on the policy fellowship schemes to step back and review the process and their questions so far and highlight areas where they might consider modifying their approaches. 10 senior policy fellows agreed to take part, 3 women and 7 men, five were from the CSaP fellowship scheme and 5 were from the CAPE fellowship scheme.

Below is an overall summary of the response that the 10 senior policy professionals gave to the 5 questions they were presented. Two independent coders reviewed the responses to all questions that are presented in more detail at the end of this section. Rather than develop a specific coding frame, each coder listed the most common types of response, and the final compiled list presented here reflects the essential properties the response give across all 10 interviews conducted.

Q1. In response to the question: What does a valuable exchange look like?

Summary of responses:

1. Take an exploratory approach to the exchanges (without expectations) (100%)
2. Gauging and then filtering what is relevant based on whether academics are attracted to the same issues/questions (70%)
3. finding ways of accessing relevant expertise (70%)

[7] https://www.cape.ac.uk/2022/05/31/roshnee-patel-fellowship/
[8] https://www.cape.ac.uk/cape-case-study-richard-whittle-policy-fellow-january-2022/

4. find some solutions to problems (30%)
5. attract interest from academics to the issues/dept that they work in (40%)
6. Expanding the range of expertise/advice available that might have been overlooked (70%)

Q2. In response to the question: What do think motivates an academic in this exchange?

Summary of responses:

1. Understand how their research could connect to a policy area (40%)
2. Understand better how policy making works (30%)
3. Highlight some of the bigger picture issues that might be missed by policy (30%)
4. Gain a better idea of what policy is currently concerned with (40%)
5. Corrective element – by highlighting evidence/theory/methodologies that have been challenged by them (60%)
6. Foot in the door to make connections in the policy world (70%)
    1. Find ways to access funding (40%)
    2. Find ways of demonstrating impact (40%)

Q3. In response to the question: How honest can you be about some of the issues you face?

Summary of responses:

1. They can be frank (80%)
2. Rest say that they have to self-censor in some way because they are:
    1. cautious because while they might want to speak about particular issues, the policy is shared across gov. dept so they end up self-censoring (40%)
    2. Cautious about mentioning live examples because of possible controversies – media/public attention (30%)
    3. Cautions because the policy/issue is sensitive (30%)
3. Talk around examples – mostly ones from the past rather than current live examples (40%)
4. Refer to common standard problems rather than specific issues/or specific illustrations (50%)
5. work with hypotheticals to get round issues of sensitivity (20%)

Q4. In response to the question: What informed the way in which you formulated your questions?

Summary of responses [9]:

1. Don’t use questions as a script, so they are just there in a way to indicate general interest in topics (60%)
2. Informed by the dept/team that they work with/oversee
    1. - in some way instructed to find specific solutions to problems* (30%)
3. Sense checked the questions against an academic first (40%)
4. Developed them in a way to be deliberately open (80%)
5. Developed them to be highly specialised (40%)

Q5. In response to the question: What is it that you can learn/have learnt from your exchanges?

Summary of responses:

1. Stock take of where they are at career wise (30%)
2. Think about the “bigger picture” and what to prioritise (60%)
3. Challenge the way they/organisation consider ways of addressing policy issues/see advice (50%)
4. Identify ways of accessing best advice (70%)
5. Identify ways of implementing news schemes (50%)
6. Cross referencing/validating (30%)
    1. Contextualise understanding of policy issues and how other policy makers think about them – given their exchanges with the same academics in the network (20%)

---

[9] 70% revised their questions, either while they started their fellowship, or as a result of the “pilot”, for those that revised them some of the issues were because academics gave highly specialised responses, but not ones that had any practical applications, - so responses were too technical, and too narrow, - so the dominant shift was to revise the questions to make them more open.

Comprehensive details of responses to the interview questions (details were omitted in the responses that could be used to identify the respondents).

Interview 1: Civil Servant {woman} (CSAP)

> Constructive-deconstruction element 1 [intentions-self]: The starting point is to work backwards by encouraging the policy fellows to consider what the anticipated outcomes of the exchanges with academics are and how realistic they are (e.g. What would a valuable exchange be for you? What might be a less valuable exchange?).

No expectations for the exchanges. This is my second fellowship at CSaP whereas before I was asking questions that were solution focused, the questions are deliberately open, so I want to explore any avenue that is taken, so I have avoided setting any expectations from any of the exchanges.

> Constructive-deconstruction element 2 [intentions-other]: The next stage is to also consider what the interests are of those in the exchange. Here the policy fellow will be invited to consider what the interests and motivations of the academic might be in an anticipated exchange (e.g. What do you think an academic might what to know from you? What kind of things do you think an academic might be interested in telling you about?).

Again, from the experiences so far, because I've left things open, we have gone through different avenues, so some have talked about their area of expertise how it connects to my policy domain, others have talked about their views on the bigger picture issues that are in my questions. There might be things that could be followed up, but I would do so unless I there is something tangible as I would be conscious of wasting people's time.

> Deconstructive element 1 [challenges]: The aim here is to help expose what the specific exchanges with academics could be that present possible challenges (e.g. Is there an opportunity to discuss with academics the challenges you face around the policy issue you are interested in? How honest can you be about to the barriers and practicalities to addressing the policy issue you are considering during your fellowship?).

I can be frank because this is an opportunity for me to learn and take a different direction, so I can offer my views [because they are speaking on behalf of themselves].

> Deconstructive element 2 [goals]: The aim here is to encourage the policy fellow to consider the underlying views and assumptions that underpin the way they are thinking about the policy issue (e.g. What is the starting point of the policy issue and what assumptions does it depend on? How did this influence the way the policy questions were formed that you proposed as part of your fellowship?).

I am willing to do this, I think what I take from this is that I really should think about what my world view is, and inspect that and think about where that is coming from and why.

> Constructive element 2 [goals]: The final component is to examine whether there are ways in which the types of inquiries that policy fellows are considering as part of their fellowship could be adapted (e.g. What is it that you really need to know? What are you most interested in finding out and learning from?).

Using the fellowship – to take time out and think and change perspectives – what kind of career and how to approach questions of the day. I am asking the big picture questions because it is a launching point to think about big issues, but also think about what I want to priortise and focus on

---

Interview 2: Civil Servant {man} (CAPE)

> Constructive-deconstruction element 1 [intentions-self]: The starting point is to work backwards by encouraging the policy fellows to consider what the anticipated outcomes of the exchanges with academics are and how realistic they are (e.g. What would a valuable exchange be for you? What might be a less valuable exchange?).

There are two ways I would like to benefit from the fellowship, and that is to learn about what available expertise there is, and also get specific help on some of specific issues that I am currently tackling. Though some of my thinking has had to change from the time I set up the questions are my role has changed, but even so there are big questions I would like to get some insight into. Such as are there any trends in the kinds of policy issues historically, and also what are ways in which we understand behaviour and how behaviour can be changed. I can see how it might be useful to ask which if any of the questions the experts had in mind to answer, of the experts were generally interesting in a discussion around the topic to get a dialogue going. I don’t want to limit what I get out of this.

> Constructive-deconstruction element 2 [intentions-other]: The next stage is to also consider what the interests are of those in the exchange. Here the policy fellow will be invited to consider what the interests and motivations of the academic might be in an anticipated exchange (e.g. What do you think an academic might what to know from you? What kind of things do you think an academic might be interested in telling you about?).

Some probably want access to funding, and some might want to be able to communicate their research, or else to get an idea of what policy issues the civil service is currently dealing with. From my part I would like to what the focus of the topics are that academic experts are interested in addressing. They might also want to find ways to be able to access ways to be involved more directly in discussions early on in the kinds of things we do.

> Deconstructive element 1 [challenges]: The aim here is to help expose what the specific exchanges with academics could be that present possible challenges (e.g. Is there an opportunity to discuss with academics the challenges you face around the policy issue you are interested in? How honest can you be about to the barriers and practicalities to addressing the policy issue you are considering during your fellowship?).

I can give examples of some real issues we are dealing with, and I know I have to self-censor some of the details, but there ways in which I can now revaluate what needs to be said to provide a clearer picture of issues or a specific problem in a way that the basics are more clearly understood. Also having thought of some examples now, it is clear to me also how I could have used the fellowship get some advice and incorporate the learnings into evaluation reports about the schemes we have trialled, and where recommendations for improvements in the future can be made.

> Deconstructive element 2 [goals]: The aim here is to encourage the policy fellow to consider the underlying views and assumptions that underpin the way they are thinking about the policy issue (e.g. What is the starting point of the policy issue and what assumptions does it depend on? How did this influence the way the policy questions were formed that you proposed as part of your fellowship?).

Civil servants end up moving across teams and sometimes depts. so I not all of the questions are now the ones that I think would be questions I want to include. It will start to think about revising some of the questions with a view to how the fellowship can be useful for my future role, and I'm currently learning about what things I will need to do. I can also see now that some of the questions are very long and overly specific and that might put people off attempting to answer them. I am not really using all the questions as part of a script, it really is a platform to get discussions going and I can see how I can use them as topic guides rather than try to get answers to very precise questions. And yes, as you pointed out, It might be worth me giving a bit of context for what the reasoning is behind the questions, so the experts have a better idea where I am coming from.

> Constructive element 2 [goals]: The final component is to examine whether there are ways in which the types of inquiries that policy fellows are considering as part of their fellowship could be adapted (e.g. What is it that you really need to know? What are you most interested in finding out and learning from?).

I want to leave open the possibility of learning new things, and yes some of it isn't directional or solutions led based discussions, it's a way to think about how I can use different expertise and also to think more broadly about things. I do have intuitions about what areas we work on could work better and can spell out how those might be informed by evidence from different types of expertise. I also see that I don't need to swot up before I meet with each expert, and use the opening introduction to me, and for them to give me a bit of background as the launching pad to talk around the questions, and then see if there are any they want to specifically address. I can change tact based on each exchange I have.

Interview 3: Civil Servant {woman} (CAPE)

> Constructive-deconstruction element 1 [intentions-self]: The starting point is to work backwards by encouraging the policy fellows to consider what the anticipated outcomes of the exchanges with academics are and how realistic they are (e.g. What would a valuable exchange be for you? What might be a less valuable exchange?).

I would like to know how to help better utilise the expertise that is out there, as I think there is so much that we [civil service] could benefit from, and could do a lot better about how we know find it, and utilise it. It will generally be of use, as I am here to learn. I can see through this discussion that it might also be worth considering more proactively the range of domain expertise that I get a chance to speak to during the fellowship, as I might be missing out on expertise that may not immediately be obviously directly related to the policy domain that I work in.

> Constructive-deconstruction element 2 [intentions-other]: The next stage is to also consider what the interests are of those in the exchange. Here the policy fellow will be invited to consider what the interests and motivations of the academic might be in an anticipated exchange (e.g. What do you think an academic might what to know from you? What kind of things do you think an academic might be interested in telling you about?).

Most likely they want to find out how they can contribute, and where we might not be doing things quite so well, and where they might be of use, as well as all the other things, like access to funding and impact. There is probably a lot of focus on impact, and their need to demonstrate impact.

> Deconstructive element 1 [challenges]: The aim here is to help expose what the specific exchanges with academics could be that present possible challenges (e.g. Is there an opportunity to discuss with academics the challenges you face around the policy issue you are interested in? How honest can you be about to the barriers and practicalities to addressing the policy issue you are considering during your fellowship?).

There is good opportunity to be honest, for some issues that I would like help with and some insights and thinking around they are already in the news, so it isn't like I need to censor what I say. Some of the issues are public knowledge, and other issues are perennial problems. Though one issue is that we share policy with other departments, so I have to be mindful that what advice or expertise I'm getting isn't conflicting with elements that aren't under my remit, there is a lot of complexity in where it is appropriate and what experts I could bring.

> Deconstructive element 2 [goals]: The aim here is to encourage the policy fellow to consider the underlying views and assumptions that underpin the way they are thinking about the policy issue (e.g. What is the starting point of the policy issue and what assumptions does it depend on? How did this influence the way the policy questions were formed that you proposed as part of your fellowship?).

There are a lot of live problems we are dealing with right now, and there is a lot of specific things we do with getting some expert advice for, so this is a starting point, the other is that I am really passionate about the policy domain I work in, so it would be really good to take this chance to tap into what is out there are. I got some advice about to develop and craft the questions from a friend of mine that is an academic, but I can see through the examples we worked from that it might be good to rethink if I want to ask instrumental questions, or more probing questions.

I can see how it might be that some of the terms I include can be misinterpreted, so there are now ways I can think about asking what the experts take the terms to mean when they are

answering the questions. And, I can also understand that I don't necessarily need to radically revise the questions, but instead use the questions to probe further their understanding and what they take my understanding to be.

> Constructive element 2 [goals]: The final component is to examine whether there are ways in which the types of inquiries that policy fellows are considering as part of their fellowship could be adapted (e.g. What is it that you really need to know? What are you most interested in finding out and learning from?).

I know that I don't want to have solution focused exchanges, this is an open exchange, and I want to maximize the opportunities that I get from these exchanges to I know where I might be able to get the best advice in the future.

Interview 4: Civil Servant, {woman} (CAPE)

> Constructive-deconstruction element 1 [intentions-self]: The starting point is to work backwards by encouraging the policy fellows to consider what the anticipated outcomes of the exchanges with academics are and how realistic they are (e.g. What would a valuable exchange be for you? What might be a less valuable exchange?).

I'm solutions oriented, and very pragmatic as a personality anyway. So I like to get specific outcomes. So the exchanges I have found that worked, and that I am most looking forward to are with academics that are knowledgeable about the specific areas that I am concerned with, I really what to get ideas for how to address them, and what evidence is out there. The flip side is that those that don't really know much about the specific policy areas, and have general interests is o.k. but this wouldn't work so well as an exchange. Also, the while the whole fellowship process is great, I wish I had more time, the process is quite intense, as I meet with academics back to back in a day, and there is a lot to digest, and then I have to go back to the day job, or move on to my next placement. So having more time to make the most of the exchanges would be good, so I can process what I'm learning.

> Constructive-deconstruction element 2 [intentions-other]: The next stage is to also consider what the interests are of those in the exchange. Here the policy fellow will be invited to consider what the interests and motivations of the academic might be in an anticipated exchange (e.g. What do you think an academic might what to know from you? What kind of things do you think an academic might be interested in telling you about?).

I think that the they are viewing these exchanges are mutually beneficial, they ge to send me papers from their work, and they get to learn about a policy area, and how policy works. I get a chance to test out policy ideas with those that have expertise in the area. –(when prompted –….academics might want to use the exchanges as a mechanism for impact, or for access to funding)

> Deconstructive element 1 [challenges]: The aim here is to help expose what the specific exchanges with academics could be that present possible challenges

(e.g. Is there an opportunity to discuss with academics the challenges you face around the policy issue you are interested in? How honest can you be about to the barriers and practicalities to addressing the policy issue you are considering during your fellowship?).

I can work with scenarios, this is what I have been doing. Something like "hypotheticals" where I say, imagine if … and then ask questions around that. I don't give any hints. I also am careful as I don't want to reveal anything that is not been made public. The hypotheticals are enough, and I don't think this presents any real barrier to how the academics can help, as I think I give enough, and so far it seems o.k.

Deconstructive element 2 [goals]: The aim here is to encourage the policy fellow to consider the underlying views and assumptions that underpin the way they are thinking about the policy issue (e.g. What is the starting point of the policy issue and what assumptions does it depend on? How did this influence the way the policy questions were formed that you proposed as part of your fellowship?).

I came up with the questions because I was able to identify what the knowledge gaps are, and especially as there the policy area is new, and it hasn't been fully formulated yet, so this is where the policy fellowship is really helpful. I also did research to help construct the questions, I asked people in my policy team and that is how I constructed my questions.

Constructive element 2 [goals]: The final component is to examine whether there are ways in which the types of inquiries that policy fellows are considering as part of their fellowship could be adapted (e.g. What is it that you really need to know? What are you most interested in finding out and learning from?).

As I said, I'm tackling a new policy area, and any insights I can get that can help with developing it, and ideas for how we implement different schemes would be really good. I'm using this fellowship to address knowledge gaps, and I'm open to different perspectives, and what research is out there in tackling the kinds of issues I'm specifically concerned with.

Interview 5: Policy Professional {man} (CSaP)

Constructive-deconstruction element 1 [intentions-self]: The starting point is to work backwards by encouraging the policy fellows to consider what the anticipated outcomes of the exchanges with academics are and how realistic they are (e.g. What would a valuable exchange be for you? What might be a less valuable exchange?).

I would like to get advance on trying to understand the current educational climate in the UK. I also want to do what the future direction is of higher education, and to use the time to explore the different ways in I can realise some of the plans that I have and what the best approach is. I'm open. I want to hear the different approaches there are to understanding what I should pursue and also where I should be going. I am using this time to reconsider what the best approach is and also to test out different views.

All exchanges will be valuable. I don't want to limit what I hear from the experiences I have with talking to people, the different angles are a way to refocus what my goals are, or to make them concrete.

> Constructive-deconstruction element 2 [intentions-other]: The next stage is to also consider what the interests are of those in the exchange. Here the policy fellow will be invited to consider what the interests and motivations of the academic might be in an anticipated exchange (e.g. What do you think an academic might what to know from you? What kind of things do you think an academic might be interested in telling you about?).

I've not really thought about this, I'm hoping that my experiences and interests chime with what academics are also interested in, especially because of the issues I'm contending with to do with higher education. Not only that, I think that there is opportunity here to understand the various issues that academics face to do with the funding landscape, what skills we are training students in, how to think about impact, and what the future of society looks like. These are broad and fundamental issues that I think will be of interest for those I am talking with. I'm willing to take on board the expertise that I have opportunity to be exposed to. Hopefully there is something of value to those I am speaking with as well, so it is a two-way exchange.

> Deconstructive element 1 [challenges]: The aim here is to help expose what the specific exchanges with academics could be that present possible challenges (e.g. Is there an opportunity to discuss with academics the challenges you face around the policy issue you are interested in? How honest can you be about to the barriers and practicalities to addressing the policy issue you are considering during your fellowship?).

I'm being completely honest. I don't think this can work if I don't lay out the things I'm considering. I'm facing a lot of challenges which I want input on, and I've been fairly upfront about them because I genuinely am open to taking on board the different viewpoints I will be getting.

> Deconstructive element 2 [goals]: The aim here is to encourage the policy fellow to consider the underlying views and assumptions that underpin the way they are thinking about the policy issue (e.g. What is the starting point of the policy issue and what assumptions does it depend on? How did this influence the way the policy questions were formed that you proposed as part of your fellowship?).

The starting point is a dilemma. Which way do I go, not only regarding the core themes I am exploring in this fellowship, but also more generally. I am at a crossroads, and this is the opportunity to think through the challenges and options carefully and then invest my efforts. This is why I'm open to listening to so many views and collating the expertise from those I am getting to speak with. The questions are all based around the real issues that I need to know about. But they are the starting point for a discussion. Not the end point. I want to use them as a platform to discuss ideas.

> Constructive element 2 [goals]: The final component is to examine whether there are ways in which the types of inquiries that policy fellows are considering as

part of their fellowship could be adapted (e.g. What is it that you really need to know? What are you most interested in finding out and learning from?).

To help me think about what I really need to do. This is ongoing. There are big challenges, and exciting ones. Some have a strategic element, but there is more than just getting solutions. I want to take this time to really know where I am at and ask myself big questions.

Interview 6: Policy Professional. {man} (CAPE)

Constructive-deconstruction element 1 [intentions-self]: The starting point is to work backwards by encouraging the policy fellows to consider what the anticipated outcomes of the exchanges with academics are and how realistic they are (e.g. What would a valuable exchange be for you? What might be a less valuable exchange?).

Primarily the aim is to understanding leadership and decision-making, and help with advice for ministers. The idea would be to get a better idea of the differences as well as similarities at high level decision making between those working in the private as well as public sector. My interest is to start open ended discussions; I want to know about areas that could be useful to me, that at least was how I started off. But, that said, I am now looking to be a more focused, not solution led, more that the discussions provide something that is of practical value, that there are practical tools – or actions that I can take away. Things that I can take into conversation with people that will use what I've learnt – that are evidence lead, non-academic.

Constructive-deconstruction element 2 [intentions-other]: The next stage is to also consider what the interests are of those in the exchange. Here the policy fellow will be invited to consider what the interests and motivations of the academic might be in an anticipated exchange (e.g. What do you think an academic might what to know from you? What kind of things do you think an academic might be interested in telling you about?).

What I do is in service to the government, but essentially conduit into government. That is what I can help do is be the means by which data and research can be used in into government and civil service. Also, there are things that my experience, and my understanding of how different organisations operate that would hopefully be useful to the academics that I talk with.

Deconstructive element 1 [challenges]: The aim here is to help expose what the specific exchanges with academics could be that present possible challenges (e.g. Is there an opportunity to discuss with academics the challenges you face around the policy issue you are interested in? How honest can you be about to the barriers and practicalities to addressing the policy issue you are considering during your fellowship?).

Yes, what I deal with is of a sensitive nature, ministers don't want to be seen to not be good at their job, so we can say what we do, but not who we help, and we promise

confidentiality, we act as trusted friend. This meant that there is a process of self-censorship going on as I am having exchanges with academics. That doesn't limit the value of what I get back because there are general issues that cut across the different examples that I handle that doesn't require me having to provide specific details of who and what.

> Deconstructive element 2 [goals]: The aim here is to encourage the policy fellow to consider the underlying views and assumptions that underpin the way they are thinking about the policy issue (e.g. What is the starting point of the policy issue and what assumptions does it depend on? How did this influence the way the policy questions were formed that you proposed as part of your fellowship?).

Actually I have revised my questions. Currently the questions I've put to academics has been revised. This is the second round of questions, and based on our discussions I'm going to revise them again. The reason for considering revising them the first time around was that this was based on my first placements. It was informed by a preliminary cape meeting with Nottingham. It became clear to me that I should be less interested in overviews of how governments work, and processes, and comparative studies. While this is interesting, what I realised is that I wanted something that would be of extra benefit – more practical, how organisations work, - with scenarios – (how do you deal with time pressure), decision-making – fundamental core responsibility, so how minister make decision-making, and help through how to approach that I face uncertainty how, as a minister make a decision, and making – decisions – imperfect information. All of this is much more valuable to me, and so I revised my questions based on my initial discussions, and now will think about changing them again, by refining what I need to get out of the answers to the questions.

> Constructive element 2 [goals]: The final component is to examine whether there are ways in which the types of inquiries that policy fellows are considering as part of their fellowship could be adapted (e.g. What is it that you really need to know? What are you most interested in finding out and learning from?).

I think these exchanges are slightly transactional, they are mostly informed by academic thinking, but at the end the idea is for me to get access to things that will be of practical use to me.

Interview 7: Civil Servant. {man} (CSaP)

> Constructive-deconstruction element 1 [intentions-self]: The starting point is to work backwards by encouraging the policy fellows to consider what the anticipated outcomes of the exchanges with academics are and how realistic they are (e.g. What would a valuable exchange be for you? What might be a less valuable exchange?).

Primarily the aim is to use the connections I have made to explore more concrete outcomes. This is why I want to use this as a way to revise my questions. My previous

experiences have been extremely valuable, but now I don’t just want to open my horizons, I also need to explore some solutions oriented outcomes.

> Constructive-deconstruction element 2 [intentions-other]: The next stage is to also consider what the interests are of those in the exchange. Here the policy fellow will be invited to consider what the interests and motivations of the academic might be in an anticipated exchange (e.g. What do you think an academic might what to know from you? What kind of things do you think an academic might be interested in telling you about?).

I already have a good list of academics that I engaged with before, and know how I want to bring them in, especially for workshops. This would be a good way to utilise their knowledge, but also to help address some specific issues that would benefit from their research, which is what I would imagine they would be motivated in talking to me about.

> Deconstructive element 1 [challenges]: The aim here is to help expose what the specific exchanges with academics could be that present possible challenges (e.g. Is there an opportunity to discuss with academics the challenges you face around the policy issue you are interested in? How honest can you be about to the barriers and practicalities to addressing the policy issue you are considering during your fellowship?).

Yes, there are things that are sensitive, and so I would be bringing in experts to meet with my team so that they get a better understanding of the situations we are dealing with. But for some of the high level issues there aren’t any problems regarding sensitivity.

> Deconstructive element 2 [goals]: The aim here is to encourage the policy fellow to consider the underlying views and assumptions that underpin the way they are thinking about the policy issue (e.g. What is the starting point of the policy issue and what assumptions does it depend on? How did this influence the way the policy questions were formed that you proposed as part of your fellowship?).

I have set out five or so themes, and within them there are 2 or 3 questions. Most of the themes have been generated by different teams that have set specific tasks for me so that I use this year of the fellowship to address specific needs from the various teams. This means that while I want to learn more and use this so that I can increase my understanding and talk to a wide range of experts, I also have a reason to ensure that this part of the fellowship brings back specific outcomes. Tanglibles.

> Constructive element 2 [goals]: The final component is to examine whether there are ways in which the types of inquiries that policy fellows are considering as part of their fellowship could be adapted (e.g. What is it that you really need to know? What are you most interested in finding out and learning from?).

I am to use this as part of my own reflective piece so that I can document my learnings, the different motivations and interests have changed over the course of the fellowship and so the questions have also been adapted, it is good that I have had a chance to

change them, and that means what I need to take away from the fellowship now is different to where I was when I first started.

Interview 8: Civil Servant. {man} (CSaP)

> Constructive-deconstruction element 1 [intentions-self]: The starting point is to work backwards by encouraging the policy fellows to consider what the anticipated outcomes of the exchanges with academics are and how realistic they are (e.g. What would a valuable exchange be for you? What might be a less valuable exchange?).

I learned from my first year of my fellowship that the value of the interactions with academics is to keep things open, so I get to hear about what their interests are, rather than focus on specifics that treat the questions like a script for me to work off. The outcomes are to explore the range of advice and research interests out there, and think more broadly about how it can be utilised in my organisation.

> Constructive-deconstruction element 2 [intentions-other]: The next stage is to also consider what the interests are of those in the exchange. Here the policy fellow will be invited to consider what the interests and motivations of the academic might be in an anticipated exchange (e.g. What do you think an academic might what to know from you? What kind of things do you think an academic might be interested in telling you about?).

Most of the academics I spoke to in my first year, and those I am speaking with in my second year are interested in advising my on my thinking about their subjects. I think that they appreciate that they have a chance to steer away from areas where research suggests that the thinking is not quite right. I think they like this, and I have learnt a lot from this, as I feel I am getting a good sense of what is out there that can be used, that is cutting edge.

> Deconstructive element 1 [challenges]: The aim here is to help expose what the specific exchanges with academics could be that present possible challenges (e.g. Is there an opportunity to discuss with academics the challenges you face around the policy issue you are interested in? How honest can you be about to the barriers and practicalities to addressing the policy issue you are considering during your fellowship?).

Because I have refocussed the questions in ways that keeps things pretty broad, the aim is to give the time to those I meet to tell me about areas they are interested in and researching on. Yes there are sensitive issues that I deal with, but they don’t present a problem in how I use the fellowship. If there is opportunity for follow ups where I need to provide more details that might be sensitive, then there are mechanisms that can be used to do that, but this isn’t a problem I confronted then or now in my fellowship.

> Deconstructive element 2 [goals]: The aim here is to encourage the policy fellow to consider the underlying views and assumptions that underpin the way they are

> thinking about the policy issue (e.g. What is the starting point of the policy issue and what assumptions does it depend on? How did this influence the way the policy questions were formed that you proposed as part of your fellowship?).

I have quite a few very specific questions in my first year of my fellowship, and the focus when talking with academics was quite intense and narrow, because the questions were specific, which guided what I and the academics talked around. When I had time to reflect, I considered a different approach, which is why I changed my questions and made them more open. I didn't want to limit the types of exchanges I had, and broadening the questions, as well keeping the exchanges much looser has allowed for a wider range of ideas. I have even more to think about. The difficulty is on how to then use all this and translate it back to the day job. There are still practical matters to deal with.

> Constructive element 2 [goals]: The final component is to examine whether there are ways in which the types of inquiries that policy fellows are considering as part of their fellowship could be adapted (e.g. What is it that you really need to know? What are you most interested in finding out and learning from?).

I am adapting what I need to know based on the discussions I am having. Also, because I have a chance to meet with other policy fellows, in the discussions I have had with them, I can connect the dots. I have a sense of who they have talked to [academics] and what ideas they have drawn from, which puts in context what they are now thinking about, and which relates to what I am thinking about too.

Interview 9: Civil Servant. {man} – {joint fellowship with another from the same organisation} (CAPE)

> Constructive-deconstruction element 1 [intentions-self]: The starting point is to work backwards by encouraging the policy fellows to consider what the anticipated outcomes of the exchanges with academics are and how realistic they are (e.g. What would a valuable exchange be for you? What might be a less valuable exchange?).

Originally we had come up with questions which in hindsight weren't as good as they could have been, and this was the first experience we had with this sort of thing, so we were testing things out. The questions were a bit to open, and we were using the fellowship to look at how we could stimulate interest in academics to get involved in and offer some help in areas we are currently exploring.

> Constructive-deconstruction element 2 [intentions-other]: The next stage is to also consider what the interests are of those in the exchange. Here the policy fellow will be invited to consider what the interests and motivations of the academic might be in an anticipated exchange (e.g. What do you think an academic might what to know from you? What kind of things do you think an academic might be interested in telling you about?).

Actually we were extremely interested in this, and knowing what academics would get out of their exchanges with us. In fact one of the questions we later started to ask was ‘who do you think is interested in your research’ and the other was ‘do you think that there is too much interference from government in your research?’. The problem was that in a few situations there was a bit of defensiveness in the responses we got to these questions. It might have been that because we were asking the questions the experts weren’t as open in giving us answers that reflected what they truly thought. We were genuinely interested in knowing. Also, in my case this is part of prior work that I had been conducted as part of a bigger project. Having a deeper understanding of what experts get out of their meetings with policy is obviously of interest, but it doesn’t look like this is something that has been explored. I definitely think it should be.

Also, it didn’t also seem that the experts were necessarily interested in or comfortable talking about how their work could result in impact, again it might be because we were the ones asking questions. It seems like they were more comfortable telling us what their research was and giving detailed information about the context, rather than how it could be directly used in an impactful, in other words, practical way.

> Deconstructive element 1 [challenges]: The aim here is to help expose what the specific exchanges with academics could be that present possible challenges (e.g. Is there an opportunity to discuss with academics the challenges you face around the policy issue you are interested in? How honest can you be about to the barriers and practicalities to addressing the policy issue you are considering during your fellowship?).

We were fairly open in our exchanges, and as mentioned, we did adapt our way of interacting with experts, and some of the later visits worked better because I think some academics were more used to talking with policy people than others, which made it easier to have more free form and interesting discussions. We are limited in the way we can fund research, but there are ways in which we want to bring experts in, and this was useful experience to us, to get to know what is out there, and figure out how to tap into that expertise or combine it with the expertise we have inhouse.

> Deconstructive element 2 [goals]: The aim here is to encourage the policy fellow to consider the underlying views and assumptions that underpin the way they are thinking about the policy issue (e.g. What is the starting point of the policy issue and what assumptions does it depend on? How did this influence the way the policy questions were formed that you proposed as part of your fellowship?).

As mentioned, the initial questions we used quite open, and they were set up to identify gaps in our knowledge and evidence. The problem was that they were too open, so it ended up sounding like a “tell us about” type set up, where the experts would give us a lot of detailed and technical background to the subject areas we included in our questions. This was a bit overwhelming, and while we learnt a lot, this wasn’t what we realised we needed. What we needed was to understand how their expertise in specific areas could be used or applied in a particular way. So, we ended up revising our questions as a result. This led us to asking about things that reflected the more practical side of things, but this ended up being a problem for some as they weren’t exactly forthcoming in thinking about specific applications of their research, and were more

comfortable talking about what they were doing research wise. So, we ended up with a sort of balance between knowing more of the specifics about their research, with some general view to how it could be applied, rathe rather than knowing how it could be of practical application to the policy areas we are interested in.

> Constructive element 2 [goals]: The final component is to examine whether there are ways in which the types of inquiries that policy fellows are considering as part of their fellowship could be adapted (e.g. What is it that you really need to know? What are you most interested in finding out and learning from?).

I am not sure we got the best of the scheme, certainly not to start with because we needed to find the right fit for how to engage. We didn't set up very specific questions to start with, and so we got a lot of interesting technical background knowledge, but not something that could translate into applications or impact. What we really needed was to get to know what is out there, to address the bigger issues, bigger picture stuff, but with a view to specific applications, something practical. Once we realised that this might have something to do with how we engaged with academics we did make adjustments.

Interview 10: Civil Servant. {man} (CSaP)

> Constructive-deconstruction element 1 [intentions-self]: The starting point is to work backwards by encouraging the policy fellows to consider what the anticipated outcomes of the exchanges with academics are and how realistic they are (e.g. What would a valuable exchange be for you? What might be a less valuable exchange?).

It is a combination of things I can taken back to my organisation, as well as expanding my view on things, and also expose gaps in my understanding or challenging my understanding. I don't really want to have things I suspect confirmed, but I don't think that will happen anyway, and it hasn't happened yet. I think also, that of the exchanges I have had so far the academics are interested in knowing about what issues I am dealing with. A exchange that wouldn't work would be one where my audience aren't interested, and so far that hasn't happened.

> Constructive-deconstruction element 2 [intentions-other]: The next stage is to also consider what the interests are of those in the exchange. Here the policy fellow will be invited to consider what the interests and motivations of the academic might be in an anticipated exchange (e.g. What do you think an academic might what to know from you? What kind of things do you think an academic might be interested in telling you about?).

Actually I didn't find that academics were pushing their own work on me. The were focused, in a lot of cases, to try to find ways to answers the questions I had, but actually the questions were just a starting point rather than a script. What I noticed is that the academics had trying to find the core concepts behind my questions, and the main themes and used them to then talk about their own work. In some ways I can imagine

that this is a way to explore opportunities to know more about a policy area, but then I'm interested in exploring what academics are currently researching that is connected to my interests – so this really is a mutual exchange. As far as I see it, it is genuine sharing rather than me abstracting what I need from them.

> Deconstructive element 1 [challenges]: The aim here is to help expose what the specific exchanges with academics could be that present possible challenges (e.g. Is there an opportunity to discuss with academics the challenges you face around the policy issue you are interested in? How honest can you be about to the barriers and practicalities to addressing the policy issue you are considering during your fellowship?).

In a sense I don't see that I've self censored much, and as it is, there are enough broad cross cutting issues that I have to deal with that can be communicated in a honest manner without it presenting any issues for me or my team, or department for that matter. So I don't see any challenges, if anything the main focus for me is to be able to say what goes on and what I want some insights into so that my views can be challenged. I want to know what I don't know, and I can't achieve that if I am not open as well as receptive to what experts from academia have to say.

> Deconstructive element 2 [goals]: The aim here is to encourage the policy fellow to consider the underlying views and assumptions that underpin the way they are thinking about the policy issue (e.g. What is the starting point of the policy issue and what assumptions does it depend on? How did this influence the way the policy questions were formed that you proposed as part of your fellowship?).

There are specific issues that need to be discussed in these exchanges, and it would be good to get a range of ideas that can help steer for me, so that I can have some useful take aways. Plus I can go back to my team with avenues we can explore further, and also where we might follow up with specific academics. So this is an on-going process where the start is to use the exchanges as a springboard. There is also the grander way of thinking about the things I have to contend with, some of these are how to navigate different and changing agendas, and how thing about uncertainty and managing it. These are big broad topics, and this was something I had in mind to discuss in speaking with the experts. I wasn't sure that this was all going to happen, but I think the way in which I can access expertise though this process, just means that I can now use this in ways that I can tap into more easily.

> Constructive element 2 [goals]: The final component is to examine whether there are ways in which the types of inquiries that policy fellows are considering as part of their fellowship could be adapted (e.g. What is it that you really need to know? What are you most interested in finding out and learning from?).

Being receptive to challenge, that is a good thing to get out of this, and encountering things that are going to surprise me. Even when I have had exchanges with a little push back, I haven't really seen it as a challenge as I can appreciate their point of view, in fact at the end of the day I agree with them. But that said, it is useful to switch perspectives and that helps later down the line, as you can anticipate better where people might have views that put a different light on things.
